# Draft Genome Sequences of 1,183 *Salmonella* Strains from the 100K Pathogen Genome Project

**DOI:** 10.1128/genomeA.00518-17

**Published:** 2017-07-13

**Authors:** Nguyet Kong, Matthew Davis, Narine Arabyan, Bihua C. Huang, Allison M. Weis, Poyin Chen, Kao Thao, Whitney Ng, Ning Chin, Soraya Foutouhi, Azarene Foutouhi, James Kaufman, Yi Xie, Dylan B. Storey, Bart C. Weimer

**Affiliations:** a School of Veterinary Medicine, 100K Pathogen Genome Project, UC Davis, Davis, California, USA; b IBM Almaden Research Center, San Jose, California, USA

## Abstract

*Salmonella* is a common food-associated bacterium that has substantial impact on worldwide human health and the global economy. This is the public release of 1,183 *Salmonella* draft genome sequences as part of the 100K Pathogen Genome Project. These isolates represent global genomic diversity in the *Salmonella* genus.

## GENOME ANNOUNCEMENT

The *Salmonella* genus includes two species, *Salmonella bongori* and *Salmonella enterica*, that are subdivided into >2,500 serovars (1, 2). This organism is a persistent causative agent of gastroenteritis in humans, is carried by livestock, and is one of the most important food pathogens globally (3). Transmission occurs through the ingestion of contaminated food products, resulting in an estimated 1.4 to 1.6 million cases of salmonellosis in the United States, with an estimated cost of $2.3 billion and with ~1.5 billion cases worldwide yearly (4, 5), which results in ~1,000 deaths (6). Control strategies include increased surveillance, biocontrol, hygiene interventions, animal vaccination, and antibiotics (7), which are often used in combination with limited success to control outbreaks among humans. The common occurrence of *Salmonella* in the global food chain, combined with rapid genome evolution (8), leads to the emergence of hypervirulent and antibiotic-resistant isolates, suggesting that the genetic diversity of these organisms is circumventing current control strategies (4, 9, 10).

The 100K Pathogen Genome Project (http://www.100kgenomes.org) is a large-scale sequencing project focused on producing genomes from a worldwide consortium that represents pathogenic bacteria from the environment, plants, animals, and humans. The consortium sequenced animal- and human health-associated pathogens for use in a reference database. In this release, we sequenced *Salmonella* genomes from around the world and multiple isolation sources to better understand the genomic diversity of the genus.

All cultures from the 100K Pathogen Genome Project were collected and banked in the laboratory of Bart C. Weimer (University of California, Davis, Davis, CA). The isolates were checked for purity and stored in cryotubes. Genomic DNA was extracted from cultures grown on 1.5% Luria-Bertani agar (Difco, Franklin Lakes, NJ), lysed (11), purified with a Qiagen QIAamp DNA genomic minikit (catalog no. 51306) (12), and checked for quality (13) before fragmentation (14). Genomic DNA was fragmented using a Diagenode Biorupter or Covaris E220, and 1 µg was used for library construction with the Kapa high-throughput (HTP) library preparation kit with dual-SPRI size selection (catalog no. KK8234; Kapa Biosystems, Boston, MA) using the Agilent Bravo next-generation sequencing (NGS) workstation (Santa Clara, CA) with barcodes from Bioo Scientific NEXTflex-96 or Integrated DNA Technologies, Inc. Weimer384-TS-LT (15, 16). Producing libraries with 250- to 500-bp fragments, library amplification was done for eight cycles using the Kapa HiFi HotStart ReadyMix, followed by 1× SPRI bead cleanup. Library size was confirmed using the Agilent 2100 Bioanalyzer with the high-sensitivity DNA kit and indexed (96 genomes/lane). Indexed libraries were quantified with Kapa library quantification (catalog no. KK4824) prior to pooling, followed by sequencing on the Illumina HiSeq 2000 with PE100 (BGI@UCDavis, Sacramento, CA). Paired-end reads were assembled using SPAdes version 3.9.0 (17). In this release, the 100K Pathogen Genome Project has produced sequences for 1,183 different *Salmonella* isolates. 

### Accession number(s).

The sequences are publicly available at the 100K Project BioProject (PRJNA186441) and the NCBI GenBank accession numbers are presented in Table 1.

**TABLE 1:** Summary statistics for samples identified as *Salmonella enterica* and *Salmonella bongori*

GenBank accession no.	SRA accession no.	Isolate name	*Salmonella* species	Serotype	No. of contigs	Total genome length (bp)
MXPT00000000	SRR1840594	BCW_1508	*S. enterica*	Newport	214	5,043,101
MXPS00000000	SRR1060743	BCW_1509	*S. enterica*	Enteritidis	63	4,713,310
MXPR00000000	SRR1060742	BCW_1510	*S. enterica*	Heidelberg	55	4,711,382
MXPQ00000000	SRR1060741	BCW_1511	*S. enterica*	Typhimurium	102	4,969,983
MXPP00000000	SRR1060740	BCW_1512	*S. enterica*	Typhi	56	4,748,023
MXPO00000000	SRR1060739	BCW_1514	*S. enterica*	Hadar	59	4,670,890
MXPN00000000	SRR1814238	BCW_1515	*S. enterica*	Virchow	61	4,634,513
MXPM00000000	SRR1060738	BCW_1516	*S. enterica*	Brandenburg	203	4,619,003
MXPL00000000	SRR1060737	BCW_1517	*S. enterica*	58:l,z13,z28:z6	69	5,118,244
MXPK00000000	SRR1060736	BCW_1518	*S. enterica*	47:d:z39	134	4,989,558
MXPJ00000000	SRR1060735	BCW_1519	*S. enterica*	48:d:z6	96	4,965,789
MXPI00000000	SRR1840565	BCW_1520	*S. enterica*	50:b:z6	176	4,912,725
MXPH00000000	SRR1060734	BCW_1521	*S. enterica*	53:lz28:z39	22	4,632,667
MXPG00000000	SRR1814239	BCW_1522	*S. enterica*	39:lz28:enx	101	4,873,505
MXPF00000000	SRR1060733	BCW_1523	*S. enterica*	13,22:z29:enx	97	4,990,975
MXPE00000000	SRR1814240	BCW_1524	*S. enterica*	4,12:b:-	92	4,898,957
MXPD00000000	SRR1814241	BCW_1525	*S. enterica*	18:z4,z23:-	193	5,391,072
MXPC00000000	SRR1060732	BCW_1526	*S. enterica*	41:z4,z23:-	61	4,570,391
MXPB00000000	SRR1840566	BCW_1528	*S. enterica*	48:g,z51:-	56	4,455,491
MXPA00000000	SRR1814243	BCW_1529	*S. enterica*	21:g,z51:-	70	4,520,303
MXOY00000000	SRR1814245	BCW_1531	*S. enterica*	62:g,z51:-	92	4,553,378
MXOW00000000	SRR1060730	BCW_1534	*S. enterica*	60:r:e,n,x,z15	192	5,227,166
MXOV00000000	SRR1814247	BCW_1535	*S. enterica*	48:i:z	73	4,945,054
MXOU00000000	SRR1060729	BCW_1536	*S. enterica*	61:k:1,5,(7)	70	4,640,267
MXOT00000000	SRR1060728	BCW_1538	*S. enterica*	48:z10:e,n,x,z15	92	5,170,550
MXOS00000000	SRR1060727	BCW_1539	*S. enterica*	38:z10:z53	66	4,759,923
MXOR00000000	SRR1060726	BCW_1540	*S. enterica*	60:r:z	71	4,625,943
MXOQ00000000	SRR1814248	BCW_1541	*S. enterica*	50:i:z	73	4,832,269
MXOP00000000	SRR1814249	BCW_1542	*S. enterica*	50:g,z51:-	104	4,684,595
MXOO00000000	SRR1060725	BCW_1543	*S. enterica*	48:g,z51:-	42	4,500,926
MXON00000000	SRR1060724	BCW_1545	*S. enterica*	45:g,z51:-	68	4,627,260
MXOM00000000	SRR1060723	BCW_1546	*S. enterica*	16:z4,z32:-	65	4,622,496
MXOL00000000	SRR1060722	BCW_1547	*S. enterica*	11:z4,z23:-	61	4,821,071
MXOK00000000	SRR1814251	BCW_1548	*S. enterica*	6,7:z36:-	98	4,637,903
MXOJ00000000	SRR1814253	BCW_1550	*S. enterica*	40:g,z51:-	51	4,828,460
MXOH00000000	SRR1060721	BCW_1552	*S. enterica*	48:i:-	42	4,434,479
MXOG00000000	SRR1840568	BCW_1553	*S. bongori*	40:z35:-	68	4,619,021
MXOF00000000	SRR1060720	BCW_1554	*S. bongori*	44:z39:-	36	4,315,393
MXOE00000000	SRR1814255	BCW_1555	*S. bongori*	60:z41:-	58	4,425,408
MXOD00000000	SRR1814256	BCW_1556	*S. bongori*	66:z41:-	95	4,417,884
MXOC00000000	SRR1840569	BCW_1557	*S. bongori*	48:z35:-	36	4,444,858
MXOB00000000	SRR1060719	BCW_1558	*S. enterica*	6,14,25:z10:1,(2),7	49	4,676,575
MXOA00000000	SRR1840570	BCW_1559	*S. enterica*	11:b:1,7	80	4,583,868
MXNZ00000000	SRR1814257	BCW_1560	*S. enterica*	6,7:z41:1,7	64	4,633,237
MXNY00000000	SRR1814258	BCW_1561	*S. enterica*	11:a:1,5	43	4,644,332
MXNX00000000	SRR1814259	BCW_1562	*S. enterica*	6,14,25:a:e,n,x	110	4,721,527
MXNW00000000	SRR1060717	BCW_1564	*S. enterica*	Typhimurium	83	4,983,291
MXNV00000000	SRR1814260	BCW_1565	*S. enterica*	Typhimurium	102	5,000,922
MXNU00000000	SRR1814261	BCW_1566	*S. enterica*	Typhimurium	203	4,999,754
MXNT00000000	SRR1060716	BCW_1567	*S. enterica*	Typhimurium	70	4,992,425
MXNR00000000	SRR1060713	BCW_1570	*S. enterica*	Typhimurium	140	4,911,943
MXNQ00000000	SRR1060712	BCW_1571	*S. enterica*	Typhimurium	109	4,970,709
MXNP00000000	SRR1840571	BCW_1572	*S. enterica*	Typhimurium	238	5,018,296
MXNO00000000	SRR1840572	BCW_1573	*S. enterica*	Typhimurium	212	5,021,665
MXNN00000000	SRR1122742	BCW_1574	*S. enterica*	Typhimurium	80	4,963,252
MXNM00000000	SRR1122741	BCW_1575	*S. enterica*	Typhimurium	88	4,906,288
MXNL00000000	SRR1840573	BCW_1578	*S. enterica*	Typhimurium / DT104	122	4,959,585
MXNK00000000	SRR1060709	BCW_1580	*S. enterica*	4,[5],12:i:-	75	5,002,197
MXNJ00000000	SRR1060708	BCW_1581	*S. enterica*	4,[5],12:i:-	90	5,071,921
MXNI00000000	SRR1122739	BCW_1582	*S. enterica*	4,[5],12:i:-	69	4,892,165
MXNH00000000	SRR1060707	BCW_1583	*S. enterica*	4,[5],12:i:-	85	4,890,514
MXNG00000000	SRR1814262	BCW_1584	*S. enterica*	4,[5],12:i:-	56	4,676,580
MXNF00000000	SRR1814263	BCW_1585	*S. enterica*	4,[5],12:i:-	78	4,892,982
MXNE00000000	SRR1060706	BCW_1587	*S. enterica*	Enteritidis	37	4,724,875
MXND00000000	SRR1122738	BCW_1588	*S. enterica*	Enteritidis	33	4,732,603
MXNC00000000	SRR1122737	BCW_1589	*S. enterica*	Saphra	56	4,618,391
MXNB00000000	SRR1060705	BCW_1590	*S. enterica*	Rubislaw	38	4,536,667
MXNA00000000	SRR1060704	BCW_1591	*S. enterica*	Michigan	50	4,766,654
MXMZ00000000	SRR1060703	BCW_1592	*S. enterica*	Urbana	44	4,453,458
MXMY00000000	SRR1814264	BCW_1593	*S. enterica*	Vietnam	95	4,899,647
MXMX00000000	SRR1060702	BCW_1594	*S. enterica*	Tornow	59	4,804,520
MXMW00000000	SRR1060701	BCW_1595	*S. enterica*	Gera	34	4,642,375
MXMV00000000	SRR1122735	BCW_1597	*S. enterica*	Brisbane	41	4,821,806
MXMU00000000	SRR1122733	BCW_1599	*S. enterica*	München	24	4,632,602
MXMT00000000	SRR1814265	BCW_1600	*S. enterica*	Senftenberg	115	5,555,004
MXMS00000000	SRR1060700	BCW_1601	*S. enterica*	Muenster	25	4,633,970
MXMR00000000	SRR1060699	BCW_1602	*S. enterica*	Montevideo	51	4,742,041
MXMQ00000000	SRR1060698	BCW_1603	*S. enterica*	Johannesburg	34	4,701,550
MXMP00000000	SRR1060697	BCW_1604	*S. enterica*	Javiana	60	4,514,150
MXMO00000000	SRR1122732	BCW_1605	*S. enterica*	Inverness	31	4,887,144
MXMN00000000	SRR1060696	BCW_1606	*S. enterica*	Cubana	73	4,946,393
MXMM00000000	SRR1060695	BCW_1607	*S. enterica*	Cerro	38	4,694,753
MXML00000000	SRR1060694	BCW_1608	*S. enterica*	Alachua	44	4,757,395
MXMK00000000	SRR1814267	BCW_1629	*S. enterica*	Arizonae	54	4,603,290
MXMJ00000000	SRR1814268	BCW_1640	*S. enterica*	Arizonae	96	4,495,589
MXMI00000000	SRR1814269	BCW_1731	*S. enterica*	Thompson	51	4,446,777
MXMH00000000	SRR1814270	BCW_1733	*S. enterica*	Thompson	43	5,012,234
MXMF00000000	SRR1814271	BCW_1760	*S. enterica*	Typhimurium	103	4,939,832
MXME00000000	SRR1814272	BCW_1769	*S. enterica*	Typhimurium	84	4,864,337
MXMD00000000	SRR1814273	BCW_1776	*S. enterica*	Saint Paul	45	4,767,634
MXMC00000000	SRR1840576	BCW_1800	*S. enterica*	Paratyphi B	110	4,709,607
MXMB00000000	SRR1814274	BCW_1809	*S. enterica*	Paratyphi B	52	4,606,360
MXMA00000000	SRR1840578	BCW_1811	*S. enterica*	Paratyphi B	113	4,709,192
MXLZ00000000	SRR1840579	BCW_1812	*S. enterica*	Paratyphi B	52	4,663,216
MXLY00000000	SRR1814275	BCW_1819	*S. enterica*	München	79	4,811,983
MXLX00000000	SRR1814276	BCW_1833	*S. enterica*	Choleraesuis	59	4,658,012
MXLW00000000	SRR1840580	BCW_1834	*S. enterica*	Decateur	87	4,692,391
MXLV00000000	SRR1814277	BCW_1852	*S. enterica*	Infantis	75	4,633,326
MXLT00000000	SRR1814278	BCW_1861	*S. enterica*	München	61	4,715,517
MXLS00000000	SRR1814279	BCW_1862	*S. enterica*	Newport	61	4,829,688
MXLR00000000	SRR1840582	BCW_1863	*S. enterica*	Newport	89	4,888,597
MXLQ00000000	SRR1814280	BCW_1864	*S. enterica*	Newport	132	5,318,034
MXLP00000000	SRR1840583	BCW_1870	*S. enterica*	Paratyphi B	60	4,755,419
MXLO00000000	SRR1814281	BCW_1872	*S. enterica*	Paratyphi B	56	4,655,098
MXLN00000000	SRR1814282	BCW_1877	*S. enterica*	Pullorum	35	4,743,041
MXLM00000000	SRR1840585	BCW_1886	*S. enterica*	Stanley	50	4,785,590
MXLK00000000	SRR1814283	BCW_1908	*S. enterica*		86	4,775,306
MXLI00000000	SRR1840591	BCW_1933	*S. enterica*		62	4,889,133
MXLH00000000	SRR1840593	BCW_1941	*S. enterica*		87	4,488,115
MXLG00000000	SRR1840595	BCW_1947	*S. enterica*		92	4,682,943
MXLF00000000	SRR1840597	BCW_1951	*S. enterica*		127	4,687,494
MXLD00000000	SRR1840599	BCW_1969	*S. enterica*		47	4,536,861
MXLC00000000	SRR1814284	BCW_1975	*S. enterica*	Enteritidis	56	4,694,921
MXLB00000000	SRR1814285	BCW_1976	*S. enterica*	Berta	80	4,768,086
MXLA00000000	SRR1814286	BCW_1977	*S. enterica*	Dublin	89	5,052,403
MXKZ00000000	SRR1814287	BCW_1978	*S. enterica*	Cerro	67	4,543,024
MXKY00000000	SRR1814288	BCW_1979	*S. enterica*	Heidelberg	52	4,754,368
MXKX00000000	SRR1814289	BCW_1980	*S. enterica*	Heidelberg	48	4,748,546
MXKW00000000	SRR1814290	BCW_1981	*S. enterica*	Anatum	27	4,675,371
MXKV00000000	SRR1814291	BCW_1982	*S. enterica*		54	4,815,529
MXKU00000000	SRR1814292	BCW_1983	*S. enterica*	Enteritidis	34	4,696,993
MXKT00000000	SRR1814293	BCW_1984	*S. enterica*	Enteritidis	41	4,698,141
MXKS00000000	SRR1814294	BCW_1985	*S. enterica*	Enteritidis	38	4,696,431
MXKR00000000	SRR1814295	BCW_1986	*S. enterica*	Enteritidis	36	4,692,804
MXKQ00000000	SRR1814296	BCW_1987	*S. enterica*	Enteritidis	39	4,696,439
MXKP00000000	SRR1814297	BCW_1988	*S. enterica*	Enteritidis	41	4,805,642
MXKO00000000	SRR1814298	BCW_1989	*S. enterica*	Enteritidis	60	4,550,842
MXKN00000000	SRR1814299	BCW_1990	*S. enterica*	Enteritidis	45	4,817,009
MXKM00000000	SRR1814300	BCW_1991	*S. enterica*	Enteritidis	26	4,724,222
MXKL00000000	SRR1814301	BCW_1992	*S. enterica*	Enteritidis	29	4,792,749
MXKK00000000	SRR1814302	BCW_1993	*S. enterica*	Dublin	48	4,941,697
MXKJ00000000	SRR1814303	BCW_1994	*S. enterica*		37	4,712,333
MXKI00000000	SRR1814304	BCW_1995	*S. enterica*	Kentucky	56	4,720,714
MXKH00000000	SRR1814305	BCW_1996	*S. enterica*		39	4,714,017
MXKG00000000	SRR1814306	BCW_1997	*S. enterica*	Cerro	39	4,545,913
MXKF00000000	SRR1814307	BCW_1998	*S. enterica*		108	4,698,466
MXKE00000000	SRR1814308	BCW_1999	*S. enterica*	Montevideo	33	4,593,636
MXKB00000000	SRR1814311	BCW_2003	*S. enterica*	Menhaden	43	4,662,475
MXKA00000000	SRR1814312	BCW_2004	*S. enterica*	Give	95	4,563,009
MXJZ00000000	SRR1814313	BCW_2005	*S. enterica*		35	4,543,254
MXJY00000000	SRR1814314	BCW_2008	*S. enterica*		82	5,119,710
MXJX00000000	SRR1814315	BCW_2010	*S. enterica*	Kentucky	81	4,867,403
MXJV00000000	SRR1814317	BCW_2013	*S. enterica*	Enteritidis	42	4,704,898
MXJU00000000	SRR1814319	BCW_2015	*S. enterica*	Enteritidis	39	4,697,368
MXJT00000000	SRR1814320	BCW_2016	*S. enterica*		37	4,695,663
MXJS00000000	SRR1814321	BCW_2017	*S. enterica*	Enteritidis	43	4,692,002
MXJR00000000	SRR1814322	BCW_2018	*S. enterica*	Reading	14	4,573,738
MXJQ00000000	SRR1814323	BCW_2019	*S. enterica*	Enteritidis	55	4,698,743
MXJP00000000	SRR1814324	BCW_2020	*S. enterica*	Enteritidis	40	4,699,383
MXJO00000000	SRR1122673	BCW_2022	*S. enterica*	Newport	85	4,855,366
MXJN00000000	SRR1060585	BCW_2024	*S. enterica*	Heidelberg	71	4,979,333
MXJM00000000	SRR1060584	BCW_2025	*S. enterica*	Typhimurium var. 5-	74	4,848,531
MXJL00000000	SRR1122671	BCW_2026	*S. enterica*	Hadar	218	4,721,180
MXJK00000000	SRR1060583	BCW_2028	*S. enterica*	Agona	42	4,839,359
MXJJ00000000	SRR1122669	BCW_2029	*S. enterica*	4,[5],12:r:-	51	4,871,561
MXJI00000000	SRR1122668	BCW_2030	*S. enterica*	4,12:d:-	132	5,071,832
MXJH00000000	SRR1122667	BCW_2031	*S. enterica*	4,[5],12:r:-	64	4,865,995
MXJG00000000	SRR1814325	BCW_2032	*S. enterica*	4,[5],12:r:-	58	4,951,606
MXJF00000000	SRR1060582	BCW_2033	*S. enterica*	Schwarzengrund	40	4,808,106
MXJE00000000	SRR1060581	BCW_2034	*S. enterica*	Alachua	52	4,804,786
MXJD00000000	SRR1122666	BCW_2035	*S. enterica*	Typhimurium var. 5-	111	5,030,715
MXJC00000000	SRR1060580	BCW_2036	*S. enterica*	Typhimurium var. 5-	182	5,107,767
MXJB00000000	SRR1060579	BCW_2037	*S. enterica*	Worthington	90	5,177,860
MXJA00000000	SRR1060578	BCW_2038	*S. enterica*	Derby	74	4,905,930
MXIZ00000000	SRR1122665	BCW_2039	*S. enterica*	Enteritidis	89	4,703,711
MXIY00000000	SRR1060577	BCW_2040	*S. enterica*	Kentucky	268	4,860,286
MXIX00000000	SRR1122664	BCW_2042	*S. enterica*	Infantis	87	4,852,561
MXIW00000000	SRR1840600	BCW_2044	*S. enterica*	4,[5],12:i:-	162	4,888,737
MXIV00000000	SRR1122662	BCW_2045	*S. enterica*	4,[5],12:i:-	280	4,876,792
MXIU00000000	SRR1122661	BCW_2046	*S. enterica*	4,12:d:-	115	5,064,568
MXIT00000000	SRR1122660	BCW_2047	*S. enterica*	Oranienburg	45	4,745,797
MXIS00000000	SRR1122659	BCW_2048	*S. enterica*	Enteritidis	70	4,605,223
MXIR00000000	SRR1122658	BCW_2049	*S. enterica*	Infantis	56	4,636,349
MXIQ00000000	SRR1122657	BCW_2050	*S. enterica*	Typhimurium var. 5-	104	5,119,486
MXIP00000000	SRR1122656	BCW_2051	*S. enterica*	4,[5],12:r:-	65	4,868,669
MXIN00000000	SRR1840601	BCW_2053	*S. enterica*	Derby	85	4,835,910
MXIM00000000	SRR1060576	BCW_2054	*S. enterica*	Typhimurium var. 5-	68	4,952,695
MXIL00000000	SRR1060575	BCW_2055	*S. enterica*	Saint Paul	85	4,823,504
MXIK00000000	SRR1840602	BCW_2056	*S. enterica*	18:z4,z23:-	102	4,563,960
MXIJ00000000	SRR1122654	BCW_2057	*S. enterica*	Newport	239	4,715,388
MXII00000000	SRR1122653	BCW_2058	*S. enterica*	Schwarzengrund	140	4,908,833
MXIH00000000	SRR1122650	BCW_2059	*S. enterica*	18:z4,z23:-	269	4,981,191
MXIG00000000	SRR1122648	BCW_2064	*S. enterica*	Typhimurium var. 5-	73	4,941,624
MXIF00000000	SRR1122647	BCW_2065	*S. enterica*	Typhimurium var. 5-	84	4,952,792
MXIB00000000	SRR1060574	BCW_2070	*S. enterica*	Kentucky	85	4,865,027
MXIA00000000	SRR1060573	BCW_2071	*S. enterica*	Schwarzengrund	50	4,673,750
MXHZ00000000	SRR1060572	BCW_2073	*S. enterica*	Typhimurium var. 5-	60	4,921,867
MXHY00000000	SRR1122645	BCW_2074	*S. enterica*	Hadar	52	4,732,446
MXHX00000000	SRR1122643	BCW_2077	*S. enterica*	Albert	126	5,141,960
MXHW00000000	SRR1106607	BCW_2079	*S. enterica*	Agona	70	4,763,712
MXHV00000000	SRR1106606	BCW_2083	*S. enterica*	Derby	113	4,792,186
MXHT00000000	SRR1106605	BCW_2085	*S. enterica*	Typhimurium	116	4,865,658
MXHS00000000	SRR1060571	BCW_2086	*S. enterica*	Hadar	71	4,642,911
MXHR00000000	SRR1060570	BCW_2087	*S. enterica*	Schwarzengrund	33	4,756,339
MXHQ00000000	SRR1060569	BCW_2088	*S. enterica*	18:z4,z23:-	65	4,487,872
MXHP00000000	SRR1840603	BCW_2090	*S. enterica*	18:z4,z23:-	123	4,487,102
MXHO00000000	SRR1840604	BCW_2092	*S. enterica*	6,7:k:-	54	4,709,630
MXHN00000000	SRR1122636	BCW_2093	*S. enterica*	Kentucky	181	4,852,382
MXHG00000000	SRR1106600	BCW_2104	*S. enterica*	Anatum	79	4,698,790
MXHE00000000	SRR1122629	BCW_2112	*S. enterica*	6,7:lw:-	36	4,687,914
MXHD00000000	SRR1106598	BCW_2115	*S. enterica*	Kentucky	165	5,095,529
MXHC00000000	SRR1106597	BCW_2120	*S. enterica*	Typhimurium var. 5-	256	4,992,470
MXGZ00000000	SRR1106595	BCW_2124	*S. enterica*	Senftenberg	96	4,852,968
MXGX00000000	SRR1840612	BCW_2127	*S. enterica*	Typhimurium var. 5-	119	4,855,797
MXGV00000000	SRR1106593	BCW_2130	*S. enterica*	Berta	62	4,891,577
MXGU00000000	SRR1106592	BCW_2131	*S. enterica*	Typhimurium var. 5-	154	4,960,828
MXGT00000000	SRR1122620	BCW_2132	*S. enterica*	Albany	39	4,731,428
MXGS00000000	SRR1106591	BCW_2133	*S. enterica*	Kentucky	168	5,020,798
MXGR00000000	SRR1106590	BCW_2134	*S. enterica*	Agona	144	4,866,625
MXGP00000000	SRR1840613	BCW_2136	*S. enterica*	Saint Paul	63	4,760,305
MXGO00000000	SRR1840614	BCW_2142	*S. enterica*	Agona	261	4,884,775
MXGN00000000	SRR1840615	BCW_2146	*S. enterica*	Typhimurium var. 5-	103	5,067,207
MXGM00000000	SRR1840616	BCW_2147	*S. enterica*	Agona	117	4,857,781
MXGL00000000	SRR1840617	BCW_2152	*S. enterica*	Senftenberg	67	4,844,931
MXGK00000000	SRR1122608	BCW_2153	*S. enterica*	Kentucky	72	4,666,800
MXGJ00000000	SRR1060567	BCW_2159	*S. enterica*	Ohio	46	4,696,704
MXGH00000000	SRR1106585	BCW_2161	*S. enterica*	Kentucky	95	4,896,056
MXGG00000000	SRR1060566	BCW_2163	*S. enterica*	Typhimurium var. 5-	71	5,009,952
MXGF00000000	SRR1060565	BCW_2166	*S. enterica*	Saint Paul	70	4,775,983
MXGE00000000	SRR1060564	BCW_2167	*S. enterica*	Saint Paul	135	5,073,352
MXGD00000000	SRR1060563	BCW_2168	*S. enterica*	Agona	90	4,847,823
MXGC00000000	SRR1060562	BCW_2170	*S. enterica*	München	63	4,739,641
MXGB00000000	SRR1840620	BCW_2171	*S. enterica*	Reading	188	4,708,211
MXGA00000000	SRR1060561	BCW_2172	*S. enterica*	Typhimurium var. 5-	94	4,984,682
MXFY00000000	SRR1060560	BCW_2174	*S. enterica*	Kentucky	166	5,164,127
MXFW00000000	SRR1106583	BCW_2176	*S. enterica*	Schwarzengrund	54	4,795,405
MXFV00000000	SRR1814326	BCW_2177	*S. enterica*	Typhimurium var. 5-	86	4,995,416
MXFU00000000	SRR1122602	BCW_2178	*S. enterica*	Saint Paul	129	5,018,003
MXFT00000000	SRR1060559	BCW_2179	*S. enterica*	Saint Paul	94	4,934,599
MXFQ00000000	SRR1060557	BCW_2183	*S. enterica*	Hadar	59	4,852,413
MXFP00000000	SRR1060555	BCW_2185	*S. enterica*	Kentucky	54	4,874,084
MXFO00000000	SRR1060554	BCW_2186	*S. enterica*	Alachua	61	5,056,195
MXFN00000000	SRR1060553	BCW_2187	*S. enterica*	Typhimurium var. 5-	68	5,014,429
MXFM00000000	SRR1060552	BCW_2188	*S. enterica*	Hadar	63	4,786,573
MXFL00000000	SRR1060551	BCW_2190	*S. enterica*	Berta	43	4,880,870
MXFK00000000	SRR1060550	BCW_2191	*S. enterica*	Berta	35	4,883,573
MXFJ00000000	SRR1060549	BCW_2192	*S. enterica*	Typhimurium var. 5-	70	4,951,275
MXFI00000000	SRR1840623	BCW_2193	*S. enterica*	Mbandaka	64	4,752,709
MXFH00000000	SRR1060548	BCW_2194	*S. enterica*	Berta	66	4,819,865
MXFG00000000	SRR1060547	BCW_2195	*S. enterica*	Enteritidis	57	4,704,676
MXFF00000000	SRR1060546	BCW_2196	*S. enterica*	Typhimurium	121	4,885,303
MXFE00000000	SRR1060545	BCW_2198	*S. enterica*	Saint Paul	87	4,970,010
MXFD00000000	SRR1122599	BCW_2199	*S. enterica*	Derby	61	4,960,626
MXFC00000000	SRR1060544	BCW_2200	*S. enterica*	Kentucky	78	4,759,417
MXFB00000000	SRR1060543	BCW_2201	*S. enterica*	Infantis	41	4,707,923
MXFA00000000	SRR1060542	BCW_2202	*S. enterica*	Kentucky	65	4,914,536
MXEZ00000000	SRR1060541	BCW_2203	*S. enterica*	Brandenburg	53	5,007,057
MXEY00000000	SRR1106582	BCW_2205	*S. enterica*	Schwarzengrund	199	4,793,032
MXEX00000000	SRR1840625	BCW_2208	*S. enterica*	Litchfield	112	4,690,460
MXEW00000000	SRR1814328	BCW_2209	*S. enterica*	Hadar	66	4,769,161
MXEV00000000	SRR1840626	BCW_2210	*S. enterica*	Typhimurium	124	5,052,611
MXEU00000000	SRR1840628	BCW_2213	*S. enterica*	Mbandaka	63	4,747,779
MXES00000000	SRR1060540	BCW_2216	*S. enterica*	Kentucky	89	4,964,951
MXER00000000	SRR1840629	BCW_2217	*S. enterica*	Braenderup	156	4,696,916
MXEQ00000000	SRR1840630	BCW_2218	*S. enterica*	Typhimurium	116	4,931,438
MXEP00000000	SRR1840631	BCW_2220	*S. enterica*	Kentucky	294	5,247,031
MXEO00000000	SRR1060539	BCW_2225	*S. enterica*	Kentucky	158	4,996,872
MXEN00000000	SRR1814329	BCW_2226	*S. enterica*	Agona	65	4,876,082
MXEM00000000	SRR1814330	BCW_2227	*S. enterica*	Kentucky	125	5,013,946
MXEL00000000	SRR1060538	BCW_2228	*S. enterica*	Senftenberg	72	4,899,799
MXEK00000000	SRR1122587	BCW_2233	*S. enterica*	18:z4,z23:-	64	4,470,177
MXEJ00000000	SRR1122585	BCW_2235	*S. enterica*	Infantis	59	4,679,403
MXEI00000000	SRR1060536	BCW_2237	*S. enterica*	Infantis	47	4,595,710
MXEH00000000	SRR1122583	BCW_2238	*S. enterica*	4,12:d:-	83	5,053,556
MXEG00000000	SRR1814331	BCW_2239	*S. enterica*	Senftenberg	73	5,117,613
MXEF00000000	SRR1840632	BCW_2242	*S. enterica*	Derby	118	4,945,102
MXEE00000000	SRR1060535	BCW_2243	*S. enterica*	Typhimurium var. 5-	67	4,930,988
MXED00000000	SRR1814332	BCW_2244	*S. enterica*	Typhimurium var. 5-	76	4,884,447
MXEB00000000	SRR1060534	BCW_2247	*S. enterica*	Typhimurium var. 5-	138	5,090,978
MXEA00000000	SRR1060533	BCW_2248	*S. enterica*	Uganda	23	4,728,649
MXDZ00000000	SRR1122579	BCW_2249	*S. enterica*	Kentucky	72	4,911,929
MXDY00000000	SRR1840634	BCW_2250	*S. enterica*	Kentucky	83	4,909,888
MXDX00000000	SRR1106580	BCW_2251	*S. enterica*	Heidelberg	81	4,965,326
MXDV00000000	SRR1840635	BCW_2427	*S. enterica*	Typhimurium	149	4,988,609
MXDU00000000	SRR1118721	BCW_2428	*S. enterica*	Kentucky	274	4,876,579
MXDT00000000	SRR1840636	BCW_2429	*S. enterica*	Enteritidis	91	4,700,612
MXDS00000000	SRR1118719	BCW_2431	*S. enterica*	Reading	186	4,576,842
MXDP00000000	SRR1814401	BCW_2435	*S. enterica*	Typhimurium	59	5,050,726
MXDO00000000	SRR1814402	BCW_2436	*S. enterica*	Agona	96	4,948,449
MXDN00000000	SRR1814403	BCW_2438	*S. enterica*	Reading	46	4,572,225
MXDM00000000	SRR1118717	BCW_2439	*S. enterica*	Dublin	62	4,569,337
MXDL00000000	SRR1814407	BCW_2448	*S. enterica*	Enteritidis	28	4,723,091
MXDK00000000	SRR1840638	BCW_2462	*S. enterica*	Heidelberg	73	4,865,021
MXDI00000000	SRR1840640	BCW_2465	*S. enterica*	4,12:r:-	130	4,878,304
MXDH00000000	SRR1840641	BCW_2466	*S. enterica*	Heidelberg	88	4,749,754
MXDG00000000	SRR1840642	BCW_2467	*S. enterica*	4,[5],12:r:-	102	4,749,033
MXDF00000000	SRR1840643	BCW_2468	*S. enterica*	4,12:r:-	287	4,833,793
MXDE00000000	SRR1840644	BCW_2469	*S. enterica*	4,12:r:-	97	4,882,563
MXDD00000000	SRR1840646	BCW_2471	*S. enterica*	Heidelberg	77	4,783,615
MXDB00000000	SRR1840648	BCW_2473	*S. enterica*	Heidelberg	178	4,944,435
MXDA00000000	SRR1840649	BCW_2474	*S. enterica*	4,[5],12:r:-	171	4,831,626
MXCZ00000000	SRR1840650	BCW_2475	*S. enterica*	Heidelberg	70	4,715,089
MXCY00000000	SRR1814408	BCW_2476	*S. enterica*	4,[5],12:r:-	62	5,017,787
MXCX00000000	SRR1814409	BCW_2479	*S. enterica*	4,[5],12:r:-	76	4,849,862
MXCW00000000	SRR1814410	BCW_2480	*S. enterica*	4,[5],12:r:-	48	4,870,653
MXCV00000000	SRR1814411	BCW_2481	*S. enterica*	Kentucky	84	4,883,278
MXCU00000000	SRR1814412	BCW_2482	*S. enterica*	Kentucky	63	4,906,038
MXCT00000000	SRR1814413	BCW_2483	*S. enterica*	Kentucky	110	5,011,557
MXCS00000000	SRR1814414	BCW_2484	*S. enterica*	Kentucky	104	4,849,792
MXCR00000000	SRR1814415	BCW_2485	*S. enterica*	Kentucky	96	4,877,690
MXCQ00000000	SRR1814416	BCW_2486	*S. enterica*	Kentucky	82	4,768,009
MXCO00000000	SRR1814418	BCW_2488	*S. enterica*	Kentucky	85	4,823,147
MXCN00000000	SRR1840651	BCW_2494	*S. enterica*	Montevideo	50	4,575,737
MXCM00000000	SRR1814419	BCW_2499	*S. enterica*	Newport	48	4,693,575
MXCL00000000	SRR1814420	BCW_2503	*S. enterica*	Schwarzengrund	32	4,653,488
MXCK00000000	SRR1814421	BCW_2505	*S. enterica*	Schwarzengrund	28	4,550,113
MXCJ00000000	SRR1814422	BCW_2506	*S. enterica*	Schwarzengrund	58	4,632,186
MXCI00000000	SRR1814423	BCW_2507	*S. enterica*	Schwarzengrund	51	4,775,212
MXCH00000000	SRR1814424	BCW_2511	*S. enterica*	Saint Paul	84	4,760,198
MXCG00000000	SRR1814425	BCW_2512	*S. enterica*	Saint Paul	72	4,895,102
MXCF00000000	SRR1814426	BCW_2513	*S. enterica*	Typhimurium var. 5- (Copenhagen)	93	4,972,908
MXCE00000000	SRR1814427	BCW_2514	*S. enterica*	Typhimurium	97	4,970,108
MXCD00000000	SRR1814428	BCW_2515	*S. enterica*	Typhimurium var. 5- (Copenhagen)	89	5,076,760
MXCC00000000	SRR1814429	BCW_2516	*S. enterica*	Typhimurium var. 5- (Copenhagen)	93	4,933,358
MXCB00000000	SRR1814430	BCW_2517	*S. enterica*	Typhimurium var. 5- (Copenhagen)	113	4,989,771
MXCA00000000	SRR1814431	BCW_2518	*S. enterica*	Typhimurium	96	4,862,943
MXBZ00000000	SRR1814432	BCW_2523	*S. enterica*	Typhimurium	99	4,838,556
MXBY00000000	SRR1814433	BCW_2524	*S. enterica*	Untypeable	103	5,073,979
MXBX00000000	SRR1840652	BCW_2526	*S. enterica*	Typhimurium var. 5- (Copenhagen)	161	4,847,806
MXBW00000000	SRR1814434	BCW_2530	*S. enterica*	Typhimurium var. 5- (Copenhagen)	66	5,152,485
MXBV00000000	SRR1814435	BCW_2531	*S. enterica*	Typhimurium var. 5- (Copenhagen)	78	4,889,000
MXBU00000000	SRR1814436	BCW_2534	*S. enterica*	Hadar	67	4,746,391
MXBS00000000	SRR1814437	BCW_2538	*S. enterica*	4,[5],12:i:-	90	4,899,295
MXBR00000000	SRR1814438	BCW_2539	*S. enterica*	4,[5],12:i:-	98	4,943,844
MXBQ00000000	SRR1814439	BCW_2540	*S. enterica*	4,[5],12:i:-	99	4,995,833
MXBP00000000	SRR1814440	BCW_2541	*S. enterica*	4,[5],12:i:-	79	4,939,231
MXBO00000000	SRR1814441	BCW_2544	*S. enterica*	Enteritidis	44	4,653,716
MXBN00000000	SRR1814442	BCW_2549	*S. enterica*	Enteritidis	30	4,680,867
MXBM00000000	SRR1814443	BCW_2550	*S. enterica*	Enteritidis	19	4,635,591
MXBL00000000	SRR1814444	BCW_2551	*S. enterica*	Enteritidis	38	4,697,132
MXBK00000000	SRR1814445	BCW_2553	*S. enterica*	Enteritidis	33	4,729,148
MXBJ00000000	SRR1814446	BCW_2556	*S. enterica*	Enteritidis	33	4,728,640
MXBI00000000	SRR1814447	BCW_2557	*S. enterica*	Enteritidis	35	4,729,728
MXBH00000000	SRR1840654	BCW_2558	*S. enterica*	Enteritidis	280	4,782,602
MXBG00000000	SRR1814448	BCW_2559	*S. enterica*	Enteritidis	36	4,726,483
MXBF00000000	SRR1814449	BCW_2561	*S. enterica*	Enteritidis	52	4,727,710
MYFD00000000	SRR1814450	BCW_2566	*S. enterica*	Enteritidis	47	4,747,501
MYFB00000000	SRR1814451	BCW_2568	*S. enterica*	Enteritidis	40	4,729,733
MYFA00000000	SRR1814452	BCW_2569	*S. enterica*	Enteritidis	48	4,695,999
MYEZ00000000	SRR1814453	BCW_2570	*S. enterica*	Enteritidis	34	4,745,925
MYEY00000000	SRR1814454	BCW_2577	*S. enterica*	Enteritidis	30	4,729,817
MYEW00000000	SRR1840658	BCW_2599	*S. enterica*		99	4,714,600
MYEV00000000	SRR1840659	BCW_2600	*S. enterica*		60	4,663,760
MYEU00000000	SRR1840660	BCW_2605	*S. enterica*		148	4,939,925
MYET00000000	SRR1840662	BCW_2615	*S. enterica*	Montevideo	57	4,726,958
MYES00000000	SRR1118716	BCW_2617	*S. enterica*	Mbandaka	47	4,767,147
MYER00000000	SRR1118715	BCW_2618	*S. enterica*	4,[5],12:i:-	58	4,904,890
MYEQ00000000	SRR1118713	BCW_2622	*S. enterica*	Arizonae	88	4,657,531
MYEP00000000	SRR1118712	BCW_2623	*S. enterica*	Reading	26	4,567,211
MYEO00000000	SRR1118711	BCW_2626	*S. enterica*		57	4,959,566
MYEN00000000	SRR1118710	BCW_2627	*S. enterica*	Choleraesuis	70	4,771,646
MYEM00000000	SRR1814456	BCW_2628	*S. enterica*	Typhimurium	85	4,957,007
MYEL00000000	SRR1814457	BCW_2629	*S. enterica*		70	4,795,178
MYEK00000000	SRR1840663	BCW_2631	*S. enterica*	Abaetetuba	213	5,149,627
MYEJ00000000	SRR1814458	BCW_2632	*S. enterica*	Abaetetuba	70	4,723,483
MYEI00000000	SRR1118709	BCW_2634	*S. enterica*	Albany	67	4,897,262
MYEH00000000	SRR1118708	BCW_2635	*S. enterica*	Amager	50	4,861,879
MYEG00000000	SRR1118707	BCW_2636	*S. enterica*	Amager	135	5,314,156
MYEF00000000	SRR1118706	BCW_2637	*S. enterica*	Anatum	58	4,758,918
MYEE00000000	SRR1118705	BCW_2638	*S. enterica*	Anatum	59	4,867,006
MYED00000000	SRR1814459	BCW_2639	*S. enterica*	Anatum	75	4,952,470
MYEC00000000	SRR1814460	BCW_2641	*S. enterica*	Apapa	45	4,686,719
MYEB00000000	SRR1118703	BCW_2642	*S. enterica*	Augustenborg	112	4,720,068
MYEA00000000	SRR1118702	BCW_2643	*S. enterica*	Bardo	84	5,027,341
MYDZ00000000	SRR1118700	BCW_2645	*S. enterica*	Bareilly	52	4,735,368
MYDY00000000	SRR1814461	BCW_2646	*S. enterica*	Blockley	65	4,809,216
MYDX00000000	SRR1118699	BCW_2647	*S. enterica*	Bonariensis	71	4,711,716
MYDW00000000	SRR1814462	BCW_2649	*S. enterica*	Braenderup	65	4,676,610
MYDV00000000	SRR1814463	BCW_2650	*S. enterica*	Braenderup	68	4,841,305
MYDU00000000	SRR1118696	BCW_2652	*S. enterica*	Brandenburg	41	4,620,824
MYDT00000000	SRR1814464	BCW_2653	*S. enterica*	Bredeney	65	4,773,200
MYDS00000000	SRR1118695	BCW_2654	*S. enterica*	Cerro	64	4,563,987
MYDR00000000	SRR1118694	BCW_2655	*S. enterica*	Cerro	60	4,531,988
MYDQ00000000	SRR1118693	BCW_2656	*S. enterica*	Chandans	29	4,592,734
MYDP00000000	SRR1814465	BCW_2657	*S. enterica*	Choleraesuis	97	4,814,979
MYDO00000000	SRR1814466	BCW_2658	*S. enterica*	Choleraesuis	80	4,910,586
MYDN00000000	SRR1118692	BCW_2661	*S. enterica*	Colindale	55	4,700,842
MYDM00000000	SRR1106513	BCW_2663	*S. enterica*	Concord	86	5,075,580
MYDL00000000	SRR1106512	BCW_2664	*S. enterica*	Concord	102	5,086,582
MYDK00000000	SRR1106510	BCW_2666	*S. enterica*	Concord	64	5,233,304
MYDJ00000000	SRR1106508	BCW_2669	*S. enterica*	Corvallis	33	4,735,043
MYDI00000000	SRR1106507	BCW_2671	*S. enterica*	Derby	78	4,758,580
MYDH00000000	SRR1840664	BCW_2673	*S. enterica*	Derby	138	4,882,582
MYDG00000000	SRR1106505	BCW_2674	*S. enterica*	Derby	76	4,850,410
MYDF00000000	SRR1106504	BCW_2676	*S. enterica*	Dublin	223	4,867,078
MYDE00000000	SRR1106503	BCW_2677	*S. enterica*	Dublin	61	4,873,287
MYDD00000000	SRR1106502	BCW_2678	*S. enterica*	Elizabethville	59	4,693,262
MYDC00000000	SRR1106501	BCW_2679	*S. enterica*	Enteritidis	28	4,746,785
MYDB00000000	SRR1106500	BCW_2680	*S. enterica*	Enteritidis	44	4,750,564
MYDA00000000	SRR1106499	BCW_2681	*S. enterica*	Enteritidis	69	4,790,998
MYCZ00000000	SRR1106498	BCW_2682	*S. enterica*	Enteritidis	36	4,805,531
MYCY00000000	SRR1106497	BCW_2684	*S. enterica*	Enteritidis	35	4,692,711
MYCX00000000	SRR1106496	BCW_2685	*S. enterica*	Enteritidis	42	4,702,025
MYCW00000000	SRR1106495	BCW_2686	*S. enterica*	Enteritidis	67	4,866,604
MYCV00000000	SRR1106494	BCW_2687	*S. enterica*	Enteritidis	53	4,699,274
MYCU00000000	SRR1106493	BCW_2688	*S. enterica*	Give	58	4,667,640
MYCT00000000	SRR1106492	BCW_2689	*S. enterica*	Hadar	39	4,840,854
MYCS00000000	SRR1106491	BCW_2690	*S. enterica*	Hadar	53	4,649,581
MYCR00000000	SRR1106490	BCW_2691	*S. enterica*	Haifa	88	4,934,644
MYCQ00000000	SRR1106489	BCW_2692	*S. enterica*	Haifa	82	4,817,105
MYCP00000000	SRR1106488	BCW_2694	*S. enterica*	Havana	68	4,711,484
MYCO00000000	SRR1106487	BCW_2695	*S. enterica*	Havana	51	4,708,992
MYCN00000000	SRR1106486	BCW_2696	*S. enterica*	Havana	56	4,702,412
MYCM00000000	SRR1106485	BCW_2697	*S. enterica*	Heidelberg	55	4,855,766
MYCL00000000	SRR1106484	BCW_2698	*S. enterica*	Infantis	157	5,031,662
MYCK00000000	SRR1106483	BCW_2699	*S. enterica*	Infantis	86	4,685,641
MYCJ00000000	SRR1106482	BCW_2700	*S. enterica*	Infantis	51	4,664,083
MYCI00000000	SRR1106481	BCW_2701	*S. enterica*	Infantis	45	4,659,770
MYCH00000000	SRR1106480	BCW_2702	*S. enterica*	Infantis	78	4,944,920
MYCG00000000	SRR1106479	BCW_2703	*S. enterica*	Isangi	51	4,647,115
MYCF00000000	SRR1106478	BCW_2704	*S. enterica*	Istanbul	76	4,828,760
MYCE00000000	SRR1106477	BCW_2705	*S. enterica*	Itami	61	4,665,092
MYCD00000000	SRR1106476	BCW_2706	*S. enterica*	Javiana	38	4,618,987
MYCC00000000	SRR1106475	BCW_2707	*S. enterica*	Javiana	39	4,533,521
MYCB00000000	SRR1106474	BCW_2708	*S. enterica*	Jukestown	51	4,790,000
MYCA00000000	SRR1106473	BCW_2709	*S. enterica*	Kambole	60	4,876,616
MYBZ00000000	SRR1106472	BCW_2710	*S. enterica*	Kedougou	54	4,768,433
MYBY00000000	SRR1106471	BCW_2711	*S. enterica*	Kedougou	200	4,821,733
MYBX00000000	SRR1106470	BCW_2712	*S. enterica*	Kedougou	99	4,770,857
MYBW00000000	SRR1106469	BCW_2713	*S. enterica*	Kedougou	85	4,766,820
MYBV00000000	SRR1840665	BCW_2714	*S. enterica*	Kedougou	264	4,766,326
MYBT00000000	SRR1106467	BCW_2716	*S. enterica*	Kentucky	48	4,801,445
MYBS00000000	SRR1106466	BCW_2718	*S. enterica*	Kentucky	56	4,808,879
MYBR00000000	SRR1106465	BCW_2719	*S. enterica*	Kentucky	45	4,663,128
MYBP00000000	SRR1106464	BCW_2721	*S. enterica*	Kentucky	71	4,818,522
MYBO00000000	SRR1106463	BCW_2722	*S. enterica*	Kentucky	65	4,818,813
MYBN00000000	SRR1106462	BCW_2723	*S. enterica*	Litchfield	56	4,737,205
MYBM00000000	SRR1106461	BCW_2724	*S. enterica*	Litchfield	57	4,680,881
MYBL00000000	SRR1106460	BCW_2725	*S. enterica*	London	66	4,484,878
MYBK00000000	SRR1106459	BCW_2726	*S. enterica*	Manhattan	41	4,598,525
MYBJ00000000	SRR1106458	BCW_2727	*S. enterica*	Mbandaka	52	4,691,795
MYBI00000000	SRR1106457	BCW_2728	*S. enterica*	Mbandaka	73	4,788,764
MYBH00000000	SRR1106456	BCW_2729	*S. enterica*	Mbandaka	79	4,829,236
MYBG00000000	SRR1106455	BCW_2730	*S. enterica*	Mbandaka	39	4,710,563
MYBF00000000	SRR1106454	BCW_2731	*S. enterica*	Mbandaka	47	4,707,171
MYBE00000000	SRR1106453	BCW_2732	*S. enterica*	Mgulani	78	4,671,749
MYBD00000000	SRR1106452	BCW_2733	*S. enterica*	Minnesota	58	4,503,850
MYBC00000000	SRR1106451	BCW_2734	*S. enterica*	Monschaui	100	4,655,764
MYBB00000000	SRR1106450	BCW_2735	*S. enterica*	Montevideo	53	4,750,631
MYBA00000000	SRR1106449	BCW_2736	*S. enterica*	München	292	4,863,765
MYAZ00000000	SRR1840668	BCW_2737	*S. enterica*	München	41	4,673,445
MYAX00000000	SRR1840670	BCW_2739	*S. enterica*	Muenster	66	4,740,976
MYAW00000000	SRR1840671	BCW_2740	*S. enterica*	Muenster	122	4,700,713
MYAV00000000	SRR1840672	BCW_2742	*S. enterica*	Newport	61	4,695,590
MYAU00000000	SRR1106447	BCW_2744	*S. enterica*	Newport	65	5,142,466
MYAS00000000	SRR1106446	BCW_2746	*S. enterica*	Newport	88	4,860,719
MYAN00000000	SRR1106441	BCW_2751	*S. enterica*	Oranienburg	219	5,097,962
MYAM00000000	SRR1106440	BCW_2752	*S. enterica*	Oranienburg	158	4,849,703
MYAL00000000	SRR1106439	BCW_2753	*S. enterica*	Oritamerin	81	4,621,290
MYAH00000000	SRR1106435	BCW_2757	*S. enterica*	Paratyphi B	141	4,706,118
MYAG00000000	SRR1840675	BCW_2758	*S. enterica*	Paratyphi B var. Java	59	4,702,864
MYAF00000000	SRR1106434	BCW_2759	*S. enterica*	Paratyphi B var. Java	99	5,096,972
MYAE00000000	SRR1106433	BCW_2760	*S. enterica*	Pomona	227	5,055,949
MYAD00000000	SRR1840676	BCW_2761	*S. enterica*	Poona	249	4,648,326
MYAB00000000	SRR1106431	BCW_2763	*S. enterica*	Reading	128	4,758,916
MXZY00000000	SRR1106428	BCW_2766	*S. enterica*	Rissen	175	5,003,307
MXZX00000000	SRR1814467	BCW_2768	*S. enterica*	Rubislaw	62	4,905,093
MXZU00000000	SRR1106427	BCW_2771	*S. enterica*	Saint Paul	136	4,707,964
MXZT00000000	SRR1106426	BCW_2772	*S. enterica*	San Diego	119	4,831,928
MXZS00000000	SRR1106425	BCW_2774	*S. enterica*	Schwarzengrund	64	4,834,942
MXZR00000000	SRR1840679	BCW_2775	*S. enterica*	Schwarzengrund	217	4,860,758
MXZQ00000000	SRR1106424	BCW_2776	*S. enterica*	Schwarzengrund	286	4,879,269
MXZN00000000	SRR1106422	BCW_2780	*S. enterica*	Singapore	55	4,662,807
MXZM00000000	SRR1106421	BCW_2782	*S. enterica*	Stanley	63	4,761,423
MXZL00000000	SRR1106419	BCW_2784	*S. enterica*	Stanley	244	4,736,680
MXZK00000000	SRR1106417	BCW_2786	*S. enterica*	Stanley	266	4,799,495
MXZI00000000	SRR1106416	BCW_2788	*S. enterica*	subsp. *salamae* 55:k:z39	282	4,986,449
MXZH00000000	SRR1840682	BCW_2789	*S. enterica*	Tarshyne	67	4,711,926
MXZG00000000	SRR1106415	BCW_2791	*S. enterica*	Thompson	93	4,701,530
MXZF00000000	SRR1106414	BCW_2793	*S. enterica*	Typhimurium	119	4,930,059
MXZE00000000	SRR1106413	BCW_2794	*S. enterica*	Typhimurium	66	4,656,858
MXZD00000000	SRR1106412	BCW_2795	*S. enterica*	Typhimurium	98	5,036,379
MXZC00000000	SRR1840683	BCW_2796	*S. enterica*	Typhimurium	89	4,858,717
MXZA00000000	SRR1106410	BCW_2798	*S. enterica*	Typhimurium	217	4,953,050
MXYZ00000000	SRR1840684	BCW_2799	*S. enterica*	Typhimurium	98	4,932,680
MXYY00000000	SRR1106409	BCW_2800	*S. enterica*	Typhimurium	185	4,962,429
MXYX00000000	SRR1106408	BCW_2801	*S. enterica*	Typhimurium	168	4,877,453
MXYV00000000	SRR1106405	BCW_2810	*S. enterica*	Uganda	39	4,676,926
MXYT00000000	SRR1840686	BCW_2813	*S. enterica*	Virchow	60	4,662,361
MXYS00000000	SRR1840687	BCW_2814	*S. enterica*	Virchow	65	4,754,588
MXYR00000000	SRR1840688	BCW_2815	*S. enterica*	Virchow	86	4,726,529
MXYQ00000000	SRR1840689	BCW_2816	*S. enterica*	Virchow	61	4,044,751
MXYP00000000	SRR1840690	BCW_2817	*S. enterica*	Virchow	64	4,770,834
MXYO00000000	SRR1840691	BCW_2818	*S. enterica*	Virchow	42	4,691,104
MXYM00000000	SRR1840693	BCW_2820	*S. enterica*	Weltevreden	246	4,930,717
MXYL00000000	SRR1106404	BCW_2821	*S. enterica*	Westthampton	83	4,895,392
MXYK00000000	SRR1106403	BCW_2822	*S. enterica*	Wilhemsburg	80	4,783,130
MXYJ00000000	SRR1106402	BCW_2823	*S. enterica*	Worthington	98	4,912,515
MXYH00000000	SRR1106400	BCW_2825	*S. enterica*	Typhimurium	130	4,959,161
MXYG00000000	SRR1106399	BCW_2826	*S. enterica*	Bredeney	57	4,827,769
MXYF00000000	SRR1106398	BCW_2828	*S. enterica*	Saint Paul	52	4,808,820
MXYE00000000	SRR1106397	BCW_2829	*S. enterica*	Schwarzengrund	66	4,642,170
MXYD00000000	SRR1106396	BCW_2830	*S. enterica*	Heidelberg	105	5,106,845
MXYC00000000	SRR1106395	BCW_2832	*S. enterica*	Agona	100	4,893,250
MXYB00000000	SRR1106394	BCW_2833	*S. enterica*	Dublin	57	4,965,626
MXXZ00000000	SRR1106392	BCW_2835	*S. enterica*	Kohbu	292	4,715,511
MXXY00000000	SRR1106391	BCW_2836	*S. enterica*	Typhimurium	222	4,990,088
MXXX00000000	SRR1106390	BCW_2837	*S. enterica*	Hadar	226	4,681,491
MXXV00000000	SRR1106388	BCW_2839	*S. enterica*	Derby	226	5,009,771
MXXU00000000	SRR1106387	BCW_2840	*S. enterica*	Weltevreden	158	5,062,962
MXXS00000000	SRR1106384	BCW_2843	*S. enterica*	Manhattan	155	4,619,391
MXXR00000000	SRR1106383	BCW_2844	*S. enterica*	Enteritidis	68	4,828,067
MXXQ00000000	SRR1106382	BCW_2845	*S. enterica*	Bovismorbificans	98	4,954,874
MXXO00000000	SRR1106380	BCW_2847	*S. enterica*	Paratyphi var. Java	187	4,759,782
MXXN00000000	SRR1106379	BCW_2848	*S. enterica*	Panama	178	4,878,820
MXXM00000000	SRR1057376	BCW_2849	*S. enterica*	Cerro	43	4,555,858
MXXL00000000	SRR1106378	BCW_2850	*S. enterica*	Havana	43	4,713,566
MXXK00000000	SRR1840695	BCW_2851	*S. enterica*	Vinobrady	63	4,918,375
MXXJ00000000	SRR1106377	BCW_2852	*S. enterica*	Singapore	92	4,803,394
MXXG00000000	SRR1840698	BCW_2855	*S. enterica*	Corvallis	183	4,719,825
MXXF00000000	SRR1840699	BCW_2856	*S. enterica*	Heidelberg	94	5,058,326
MXXD00000000	SRR1106375	BCW_2858	*S. enterica*	Corvallis	76	5,020,070
MXXC00000000	SRR1106373	BCW_2860	*S. enterica*	Enteritidis	70	4,805,910
MXXA00000000	SRR1840700	BCW_2862	*S. enterica*	Saint Paul	81	4,795,014
MXWZ00000000	SRR1106371	BCW_2863	*S. enterica*	Virchow	284	4,949,107
MXWY00000000	SRR1106370	BCW_2864	*S. enterica*	Rissen	115	4,995,234
MXWX00000000	SRR1815421	BCW_2865	*S. enterica*	Reading	71	4,791,130
MXWW00000000	SRR1057377	BCW_2866	*S. enterica*	London	49	4,675,757
MXWU00000000	SRR1106368	BCW_2868	*S. enterica*		200	4,950,845
MXWT00000000	SRR1106367	BCW_2869	*S. enterica*	Livingstone	147	4,875,591
MXWR00000000	SRR1106366	BCW_2871	*S. enterica*	Mbandaka	37	4,752,847
MXWP00000000	SRR1106364	BCW_2873	*S. enterica*	Poona	68	4,646,351
MXWO00000000	SRR1106362	BCW_2875	*S. enterica*	Oranienburg	55	4,510,867
MXWN00000000	SRR1106361	BCW_2876	*S. enterica*	Thompson	83	4,772,667
MXWL00000000	SRR1840702	BCW_2878	*S. enterica*	Javiana	42	4,604,457
MXWK00000000	SRR1106359	BCW_2879	*S. enterica*	Meleagridis	63	4,781,493
MXWI00000000	SRR1057378	BCW_2881	*S. enterica*	Indiana	93	4,954,921
MXWG00000000	SRR1106357	BCW_2883	*S. enterica*	Brandenburg	177	4,618,858
MXWF00000000	SRR1840704	BCW_2884	*S. enterica*	Muenster	119	4,696,579
MXWE00000000	SRR1106356	BCW_2885	*S. enterica*	Bredeney	52	4,608,344
MXWC00000000	SRR1057379	BCW_2888	*S. enterica*	Albany	67	4,800,037
MXWB00000000	SRR1057380	BCW_2889	*S. enterica*	Muenster	43	4,693,852
MXWA00000000	SRR1840705	BCW_2890	*S. enterica*	Bareilly	61	4,665,185
MXVZ00000000	SRR1106354	BCW_2891	*S. enterica*	Amsterdam	85	4,890,986
MXVY00000000	SRR1840706	BCW_2892	*S. enterica*	Litchfield	55	4,657,614
MXVX00000000	SRR1106353	BCW_2893	*S. enterica*	Senftenberg	270	4,962,330
MXVV00000000	SRR1106351	BCW_2895	*S. enterica*	Kentucky	75	4,815,133
MXVU00000000	SRR1106350	BCW_2896	*S. enterica*		91	4,868,988
MXVT00000000	SRR1106349	BCW_2897	*S. enterica*	Potsdam	216	4,885,282
MXVS00000000	SRR1106348	BCW_2898	*S. enterica*	Haifa	84	4,785,248
MXVR00000000	SRR1106347	BCW_2899	*S. enterica*	Derby	82	4,850,362
MXVP00000000	SRR1840707	BCW_2901	*S. enterica*	Onireke	81	4,815,350
MXVO00000000	SRR1057381	BCW_2903	*S. enterica*	Wippra	111	4,626,118
MXVN00000000	SRR1057382	BCW_2904	*S. enterica*	Liverpool	107	4,766,047
MXVM00000000	SRR1815423	BCW_2905	*S. enterica*	Sundsvall	71	4,585,166
MXVL00000000	SRR1815424	BCW_2906	*S. enterica*	Enteritidis	47	4,809,276
MXVK00000000	SRR1815425	BCW_2907	*S. enterica*	Eko	64	4,841,210
MXVJ00000000	SRR1057383	BCW_2908	*S. enterica*	Colindale	74	4,725,716
MXVI00000000	SRR1815426	BCW_2909	*S. enterica*	Give	57	4,688,146
MXVH00000000	SRR1815427	BCW_2910	*S. enterica*	Hillingdon	84	4,821,141
MXVG00000000	SRR1815428	BCW_2911	*S. enterica*	Stanley	44	4,723,201
MXVF00000000	SRR1815476	BCW_3047	*S. enterica*	Typhimurium	99	4,885,253
MXVE00000000	SRR1815477	BCW_3048	*S. enterica*	Enteritidis	73	4,688,373
MXVD00000000	SRR1815478	BCW_3049	*S. enterica*	Choleraesuis	70	4,736,186
MXVC00000000	SRR1815479	BCW_3050	*S. enterica*	Typhimurium	87	4,774,690
MXVB00000000	SRR1815480	BCW_3051	*S. enterica*	Typhi	66	4,600,313
MXVA00000000	SRR1815481	BCW_3052	*S. enterica*	Arizonae	43	4,476,515
MXUZ00000000	SRR1815482	BCW_3053	*S. enterica*	Newport	111	4,797,267
MXUY00000000	SRR1815483	BCW_3054	*S. enterica*	Montevideo	61	4,583,191
MXUX00000000	SRR1815484	BCW_3055	*S. enterica*		156	5,355,583
MXUW00000000	SRR1815485	BCW_3056	*S. enterica*	Typhimurium	81	4,836,687
MXUV00000000	SRR1815486	BCW_3057	*S. enterica*	Heidelberg	62	4,747,107
MXUU00000000	SRR1815487	BCW_3058	*S. enterica*	Braenderup	63	4,716,473
MXUT00000000	SRR1815488	BCW_3059	*S. enterica*	Diarizonae	85	5,132,730
MXUS00000000	SRR1815490	BCW_3061	*S. enterica*	Enteritidis	44	4,720,354
MXUR00000000	SRR1815491	BCW_3062	*S. enterica*	Typhimurium	83	4,897,532
MXUQ00000000	SRR1815492	BCW_3063	*S. enterica*	Paratyphi A	45	4,538,215
MXUP00000000	SRR1815493	BCW_3064	*S. enterica*		90	4,772,312
MXUO00000000	SRR1815494	BCW_3065	*S. enterica*		73	4,738,706
MXUM00000000	SRR1815496	BCW_3067	*S. enterica*	Anatum	51	4,665,925
MXUL00000000	SRR1815497	BCW_3068	*S. enterica*	Javiana	48	4,551,675
MXUK00000000	SRR1815498	BCW_3069	*S. enterica*	Abony	38	4,687,938
MXUJ00000000	SRR1815499	BCW_3070	*S. enterica*	Indica	24	4,693,372
MXUI00000000	SRR1815500	BCW_3071	*S. enterica*	Minnesota	36	4,546,028
MXUH00000000	SRR1815501	BCW_3072	*S. enterica*	Java	71	4,812,521
MXUG00000000	SRR1815502	BCW_3073	*S. enterica*	Abaetetuba	33	4,585,413
MXUF00000000	SRR1815503	BCW_3074	*S. enterica*	Worthington	45	4,811,099
MXUE00000000	SRR1815504	BCW_3075	*S. enterica*	Infantis	76	4,642,062
MXUD00000000	SRR1060499	BCW_3292	*S. enterica*	Bovismorbificans	29	4,840,595
MXUC00000000	SRR1815577	BCW_3293	*S. enterica*	Bovismorbificans	57	4,838,053
MXUB00000000	SRR1060498	BCW_3294	*S. enterica*	Bovismorbificans	27	4,838,851
MXUA00000000	SRR1060497	BCW_3295	*S. enterica*	Bovismorbificans	38	4,838,189
MXTZ00000000	SRR1060496	BCW_3296	*S. enterica*	Bovismorbificans	54	4,840,673
MXTY00000000	SRR1060495	BCW_3297	*S. enterica*	Bovismorbificans	45	4,844,119
MXTX00000000	SRR1815579	BCW_3299	*S. enterica*	Bovismorbificans	53	4,839,381
MXTW00000000	SRR1815580	BCW_3300	*S. enterica*	Bovismorbificans	203	4,838,812
MXTV00000000	SRR1057387	BCW_3304	*S. enterica*	Bovismorbificans	56	4,629,526
MXTU00000000	SRR1057388	BCW_3305	*S. enterica*	Bovismorbificans	47	4,566,185
MXTT00000000	SRR1057390	BCW_3307	*S. enterica*	Bovismorbificans	44	4,569,540
MXTS00000000	SRR1057391	BCW_3308	*S. enterica*	Bovismorbificans	50	4,572,698
MXTR00000000	SRR1840708	BCW_3309	*S. enterica*	Bovismorbificans	29	4,846,253
MXTQ00000000	SRR1815583	BCW_3310	*S. enterica*	Bovismorbificans	50	4,571,923
MXTP00000000	SRR1815584	BCW_3311	*S. enterica*	Bovismorbificans	73	4,746,204
MXTO00000000	SRR1815585	BCW_3312	*S. enterica*	Bovismorbificans	69	4,783,632
MXTN00000000	SRR1815641	BCW_3370	*S. enterica*	Dublin	57	4,968,344
MXTL00000000	SRR1815642	BCW_3373	*S. enterica*	Choleraesuis	173	4,732,627
MXTK00000000	SRR1815643	BCW_3376	*S. enterica*	Newport	96	4,896,385
MXTJ00000000	SRR1840711	BCW_3379	*S. enterica*	Newport	98	4,925,399
MXTI00000000	SRR1815644	BCW_3381	*S. enterica*	Newport	50	4,692,732
MXTF00000000	SRR1815646	BCW_3391	*S. enterica*	Typhimurium	156	5,129,165
MXTE00000000	SRR1122501	BCW_3397	*S. enterica*	Typhimurium	147	5,007,181
MXTC00000000	SRR1815647	BCW_3399	*S. enterica*	Typhimurium	111	4,589,713
MXTB00000000	SRR1815648	BCW_3400	*S. enterica*	Typhimurium	115	4,976,612
MXTA00000000	SRR1122500	BCW_3401	*S. enterica*	Typhimurium	100	5,006,021
MXSY00000000	SRR1815649	BCW_3403	*S. enterica*	Heidelberg	74	4,878,111
MXSW00000000	SRR1122497	BCW_3407	*S. enterica*	Heidelberg	54	4,774,229
MXSU00000000	SRR1122495	BCW_3410	*S. enterica*	Heidelberg	101	4,918,683
MXSS00000000	SRR1122493	BCW_3413	*S. enterica*	Dublin	91	4,941,315
MXSQ00000000	SRR1122491	BCW_3417	*S. enterica*	Enteritidis	40	4,697,833
MXSP00000000	SRR1122490	BCW_3418	*S. enterica*	Enteritidis	38	4,701,794
MXSO00000000	SRR1122489	BCW_3419	*S. enterica*	Newport	117	5,237,192
MXSN00000000	SRR1106560	BCW_3423	*S. enterica*	Javiana	30	4,679,164
MXSM00000000	SRR1122485	BCW_3424	*S. enterica*	Javiana	60	4,753,314
MXSL00000000	SRR1815650	BCW_3425	*S. enterica*	Javiana	78	4,597,103
MXSK00000000	SRR1840720	BCW_3429	*S. enterica*		73	4,639,209
MXSJ00000000	SRR1816009	BCW_3946	*S. enterica*	Typhimurium LT2	89	4,894,986
MXSH00000000	SRR1106341	BCW_3954	*S. enterica*	Anatum	171	4,980,508
MXSG00000000	SRR1840721	BCW_3955	*S. enterica*	Anatum	49	4,666,007
MXSF00000000	SRR1816010	BCW_3956	*S. enterica*	Javiana	48	4,629,677
MXSE00000000	SRR1106340	BCW_3957	*S. enterica*	Javiana	54	4,550,601
MXSD00000000	SRR1816011	BCW_3958	*S. enterica*	Javiana	63	4,543,778
MXSB00000000	SRR1106338	BCW_3960	*S. enterica*	München	50	4,599,061
MXSA00000000	SRR1106337	BCW_3961	*S. enterica*	München	122	5,584,917
MXRZ00000000	SRR1106336	BCW_3962	*S. enterica*	München	185	5,325,280
MXRY00000000	SRR1106335	BCW_3963	*S. enterica*	München	131	5,045,274
MXRX00000000	SRR1106334	BCW_3964	*S. enterica*	Thompson	70	4,637,934
MXRW00000000	SRR1106333	BCW_3965	*S. enterica*	Thompson	169	4,747,023
MXRV00000000	SRR1106332	BCW_3966	*S. enterica*	Thompson	131	5,080,361
MXRU00000000	SRR1106331	BCW_3967	*S. enterica*	Thompson	242	5,095,338
MXRT00000000	SRR1840722	BCW_3969	*S. enterica*	Newport (isolate S10801)	96	4,970,386
MXRS00000000	SRR1106330	BCW_3970	*S. enterica*	Anatum	77	4,659,715
MXRR00000000	SRR1106329	BCW_3971	*S. enterica*	Oranienburg	41	4,587,977
MXRQ00000000	SRR1840723	BCW_3972	*S. enterica*	Braenderup	67	4,661,629
MXRP00000000	SRR1106328	BCW_3973	*S. enterica*	Javiana	41	4,545,311
MXRO00000000	SRR1106327	BCW_3974	*S. enterica*	Infantis	96	4,603,059
MXRN00000000	SRR1816012	BCW_3975	*S. enterica*	Braenderup	151	4,654,185
MXRM00000000	SRR1840724	BCW_3976	*S. enterica*	Poona	72	4,589,727
MXRL00000000	SRR1106326	BCW_3977	*S. enterica*	Oranienburg	64	4,773,105
MXRK00000000	SRR1840725	BCW_3978	*S. enterica*	Javiana	44	4,679,302
MXRI00000000	SRR1106324	BCW_3980	*S. enterica*	Berta	55	4,751,563
MXRH00000000	SRR1106323	BCW_3981	*S. enterica*	München	86	4,813,057
MXRG00000000	SRR1106322	BCW_3982	*S. enterica*	Montevideo	137	4,737,256
MXRF00000000	SRR1106321	BCW_3983	*S. enterica*	Infantis	150	4,674,192
MXRE00000000	SRR1106320	BCW_3984	*S. enterica*	Thompson	88	4,748,262
MXRC00000000	SRR1106318	BCW_3986	*S. enterica*	Poona	57	4,607,905
MXRA00000000	SRR1106316	BCW_3988	*S. enterica*	Javiana	110	4,600,804
MXQZ00000000	SRR1106315	BCW_3989	*S. enterica*	Thompson	70	4,897,286
MXQY00000000	SRR1106314	BCW_3990	*S. enterica*	Typhimurium	78	4,828,303
MXQX00000000	SRR1840726	BCW_3992	*S. enterica*	München	49	4,733,057
MXQW00000000	SRR1816013	BCW_3993	*S. enterica*	Braenderup	69	4,696,120
MXQV00000000	SRR1816014	BCW_3994	*S. enterica*	Virchow	53	4,726,922
MXQU00000000	SRR1106312	BCW_3995	*S. enterica*	Infantis	55	4,575,526
MXQS00000000	SRR1106310	BCW_3997	*S. enterica*	Braenderup	147	4,627,535
MXQR00000000	SRR1816015	BCW_3998	*S. enterica*	Hartford	65	4,858,064
MXQQ00000000	SRR1816016	BCW_3999	*S. enterica*	Litchfield	53	4,626,170
MXQP00000000	SRR1106309	BCW_4000	*S. enterica*	Litchfield	100	4,701,917
MXQN00000000	SRR1840727	BCW_4002	*S. enterica*	Javiana	35	4,636,422
MXQM00000000	SRR1816018	BCW_4003	*S. enterica*	Thompson	90	4,702,008
MXQL00000000	SRR1106308	BCW_4004	*S. enterica*	Montevideo	211	4,726,450
MXQK00000000	SRR1106307	BCW_4005	*S. enterica*	Anatum	97	4,695,326
MXQJ00000000	SRR1106306	BCW_4006	*S. enterica*	Anatum	111	4,720,546
MYUA00000000	SRR1106303	BCW_4010	*S. enterica*	Anatum	117	4,628,901
MYTZ00000000	SRR1106302	BCW_4011	*S. enterica*	Berta	101	4,739,981
MYTY00000000	SRR1106301	BCW_4012	*S. enterica*	Hartford	153	4,847,908
MYTX00000000	SRR1106300	BCW_4013	*S. enterica*	Hartford	69	4,814,418
MYTW00000000	SRR1816019	BCW_4014	*S. enterica*	Hartford	77	4,528,757
MYTV00000000	SRR1106299	BCW_4015	*S. enterica*	Litchfield	172	4,777,346
MYTU00000000	SRR1106298	BCW_4016	*S. enterica*	Hadar	150	4,703,226
MYTT00000000	SRR1106297	BCW_4017	*S. enterica*	Paratyphi B	69	4,666,931
MYTR00000000	SRR1816050	BCW_4232	*S. enterica*	Bovismorbificans 158	43	4,725,644
MYTQ00000000	SRR1816051	BCW_4233	*S. enterica*	Choleraesuis X3264	56	4,762,068
MYTP00000000	SRR1816052	BCW_4234	*S. enterica*	Typhimurium 1408	97	4,887,615
MYTO00000000	SRR1133236	BCW_4343	*S. enterica*	Newport	144	5,176,001
MYTN00000000	SRR1133235	BCW_4344	*S. enterica*	Newport	127	4,971,739
MYTM00000000	SRR1816134	BCW_4345	*S. enterica*	Agona	71	5,133,792
MYTL00000000	SRR1133234	BCW_4346	*S. enterica*	Typhimurium var. 5-	122	4,983,525
MYTI00000000	SRR1133233	BCW_4349	*S. enterica*	Newport	205	5,112,722
MYTG00000000	SRR1133232	BCW_4351	*S. enterica*	Cubana	240	5,196,151
MYTE00000000	SRR1840731	BCW_4353	*S. enterica*	Newport	164	4,952,325
MYTC00000000	SRR1840733	BCW_4356	*S. enterica*	Enteritidis	209	4,945,394
MYTA00000000	SRR1133230	BCW_4358	*S. enterica*	Agona	89	5,032,952
MYSZ00000000	SRR1133229	BCW_4359	*S. enterica*	Dublin	51	4,974,874
MYSW00000000	SRR1133228	BCW_4362	*S. enterica*	Agona	126	5,021,755
MYST00000000	SRR1840738	BCW_4365	*S. enterica*	Typhimurium	86	5,122,219
MYSS00000000	SRR1816138	BCW_4366	*S. enterica*	Dublin	113	4,968,847
MYSQ00000000	SRR1840740	BCW_4368	*S. enterica*	Newport	72	5,015,211
MYSP00000000	SRR1840741	BCW_4369	*S. enterica*	Group C2	75	4,941,726
MYSO00000000	SRR1840742	BCW_4370	*S. enterica*	Newport	109	5,049,424
MYSN00000000	SRR1840743	BCW_4371	*S. enterica*	Newport	77	4,990,743
MYSM00000000	SRR1840744	BCW_4372	*S. enterica*	Typhimurium	121	5,150,066
MYSL00000000	SRR1840745	BCW_4373	*S. enterica*	Newport	107	4,906,835
MYSK00000000	SRR1840746	BCW_4374	*S. enterica*	Newport	76	4,977,217
MYSJ00000000	SRR1840747	BCW_4375	*S. enterica*	Agona	88	5,012,226
MYSI00000000	SRR1840748	BCW_4376	*S. enterica*	Newport	92	4,975,843
MYSH00000000	SRR1840749	BCW_4377	*S. enterica*	Agona	71	4,977,240
MYSG00000000	SRR1840750	BCW_4378	*S. enterica*	Newport	88	4,958,605
MYSF00000000	SRR1840751	BCW_4379	*S. enterica*	Typhimurium var. 5-	107	5,126,554
MYSE00000000	SRR1840752	BCW_4380	*S. enterica*	Newport	73	4,838,297
MYSD00000000	SRR1816139	BCW_4381	*S. enterica*	Typhimurium var. 5-	204	5,160,925
MYSC00000000	SRR1840753	BCW_4382	*S. enterica*	Newport	96	4,972,209
MYSA00000000	SRR1816140	BCW_4384	*S. enterica*	Heidelberg	89	5,106,196
MYRZ00000000	SRR1816141	BCW_4385	*S. enterica*	Dublin	210	4,973,427
MYRU00000000	SRR1816146	BCW_4390	*S. enterica*	Typhimurium	117	5,107,620
MYRS00000000	SRR1816148	BCW_4392	*S. enterica*	Typhimurium	142	5,126,692
MYRR00000000	SRR1816323	BCW_4651	*S. enterica*		44	4,798,821
MYRQ00000000	SRR1816324	BCW_4652	*S. enterica*		42	4,698,671
MYRP00000000	SRR1816325	BCW_4653	*S. enterica*		50	4,864,564
MYRO00000000	SRR1816327	BCW_4656	*S. enterica*		47	4,731,136
MYRN00000000	SRR1816328	BCW_4657	*S. enterica*		27	4,696,026
MYRM00000000	SRR1816329	BCW_4658	*S. enterica*		26	4,695,002
MYRL00000000	SRR1816330	BCW_4659	*S. enterica*		90	4,819,372
MYRK00000000	SRR1816331	BCW_4660	*S. enterica*		37	4,731,302
MYRJ00000000	SRR1816332	BCW_4661	*S. enterica*		43	4,694,989
MYRI00000000	SRR1816333	BCW_4662	*S. enterica*		34	4,729,771
MYRH00000000	SRR1816334	BCW_4663	*S. enterica*		37	4,696,846
MYRG00000000	SRR1816335	BCW_4664	*S. enterica*		28	4,695,186
MYRF00000000	SRR1816336	BCW_4665	*S. enterica*		44	4,697,762
MYRE00000000	SRR1816337	BCW_4666	*S. enterica*		36	4,697,934
MYRD00000000	SRR1816338	BCW_4667	*S. enterica*		32	4,637,705
MYRC00000000	SRR1816339	BCW_4668	*S. enterica*		29	4,794,897
MYRB00000000	SRR1816340	BCW_4669	*S. enterica*		46	4,703,826
MYRA00000000	SRR1816341	BCW_4670	*S. enterica*		42	4,639,441
MYQZ00000000	SRR1816342	BCW_4671	*S. enterica*		27	4,694,414
MYQY00000000	SRR1816343	BCW_4672	*S. enterica*		46	4,729,916
MYQX00000000	SRR1816344	BCW_4673	*S. enterica*		40	4,696,024
MYQW00000000	SRR1816345	BCW_4674	*S. enterica*		40	4,695,278
MYQV00000000	SRR1816346	BCW_4675	*S. enterica*		34	4,692,292
MYQU00000000	SRR1816347	BCW_4676	*S. enterica*		28	4,695,197
MYQT00000000	SRR1816348	BCW_4677	*S. enterica*		37	4,695,750
MYQS00000000	SRR1816349	BCW_4678	*S. enterica*		41	4,696,374
MYQR00000000	SRR1816350	BCW_4679	*S. enterica*		33	4,696,528
MYQQ00000000	SRR1816351	BCW_4680	*S. enterica*		40	4,698,547
MYQP00000000	SRR1816352	BCW_4681	*S. enterica*		41	4,698,290
MYQO00000000	SRR1816353	BCW_4682	*S. enterica*		35	4,704,806
MYQN00000000	SRR1816354	BCW_4683	*S. enterica*		36	4,695,392
MYQM00000000	SRR1816355	BCW_4684	*S. enterica*		47	4,529,850
MYQL00000000	SRR1816446	BCW_4834	*S. enterica*	48:-:-	98	5,163,960
MYQK00000000	SRR1816447	BCW_4835	*S. enterica*	Typhimurium	83	4,895,243
MYQJ00000000	SRR1816448	BCW_4836	*S. enterica*	Typhimurium	76	4,769,472
MYQI00000000	SRR1816449	BCW_4837	*S. enterica*	Brandenburg	61	4,446,364
MYQH00000000	SRR1816450	BCW_4838	*S. enterica*	Brandenburg	83	4,622,960
MYQG00000000	SRR1816451	BCW_4839	*S. enterica*	Typhimurium	85	4,770,466
MYQF00000000	SRR1816452	BCW_4840	*S. enterica*	Typhimurium	75	4,801,660
MYQE00000000	SRR1816453	BCW_4842	*S. enterica*	Brandenburg	52	4,629,291
MYQD00000000	SRR1816454	BCW_4843	*S. enterica*	Typhimurium	77	4,805,016
MYQC00000000	SRR1816455	BCW_4844	*S. enterica*	Typhimurium	82	4,803,735
MYQB00000000	SRR1816456	BCW_4845	*S. enterica*	Typhimurium	75	4,803,017
MYQA00000000	SRR1816457	BCW_4846	*S. enterica*	Typhimurium	94	4,894,766
MYPZ00000000	SRR1816458	BCW_4847	*S. enterica*	Typhimurium	64	4,756,875
MYPY00000000	SRR1816459	BCW_4848	*S. enterica*	Typhimurium	149	4,785,032
MYPX00000000	SRR1816460	BCW_4849	*S. enterica*	Typhimurium	78	4,846,746
MYPW00000000	SRR1816461	BCW_4850	*S. enterica*	Typhimurium	88	4,804,916
MYPV00000000	SRR1816462	BCW_4851	*S. enterica*	Typhimurium	91	4,895,455
MYPU00000000	SRR1816463	BCW_4852	*S. enterica*	Typhimurium	85	4,896,021
MYPT00000000	SRR1816464	BCW_4853	*S. enterica*	Brandenburg	25	4,531,102
MYPS00000000	SRR1816465	BCW_4854	*S. enterica*	Typhimurium	93	4,894,988
MYPR00000000	SRR1816466	BCW_4855	*S. enterica*	Typhimurium	68	4,770,991
MYPQ00000000	SRR1816467	BCW_4856	*S. enterica*	Typhimurium	95	4,845,262
MYPP00000000	SRR1816468	BCW_4857	*S. enterica*	Typhimurium	76	4,804,573
MYPO00000000	SRR1816469	BCW_4858	*S. enterica*	Typhimurium	60	4,799,354
MYPN00000000	SRR1816470	BCW_4859	*S. enterica*	Typhimurium	67	4,874,871
MYPM00000000	SRR1816471	BCW_4860	*S. enterica*	Typhimurium	62	4,768,147
MYPL00000000	SRR1816472	BCW_4861	*S. enterica*	Typhimurium	80	4,893,155
MYPK00000000	SRR1816473	BCW_4862	*S. enterica*	Brandenburg	35	4,535,313
MYPJ00000000	SRR1816474	BCW_4863	*S. enterica*	Typhimurium	84	4,802,661
MYPI00000000	SRR1816475	BCW_4864	*S. enterica*	Brandenburg	49	4,634,922
MYPH00000000	SRR1816476	BCW_4865	*S. enterica*	Brandenburg	46	4,646,741
MYPG00000000	SRR1816477	BCW_4866	*S. enterica*	Brandenburg	45	4,533,503
MYPF00000000	SRR1816495	BCW_4887	*S. enterica*		38	4,704,384
MYPE00000000	SRR1816496	BCW_4888	*S. enterica*		66	4,644,940
MYPD00000000	SRR1816497	BCW_4889	*S. enterica*		53	4,614,009
MYPC00000000	SRR1816498	BCW_4890	*S. enterica*		70	4,768,956
MYPB00000000	SRR1816499	BCW_4891	*S. enterica*		82	4,685,261
MYPA00000000	SRR1816500	BCW_4892	*S. enterica*		40	4,656,781
MYOZ00000000	SRR1816501	BCW_4893	*S. enterica*		80	4,697,252
MYOY00000000	SRR1816502	BCW_4894	*S. enterica*		35	4,663,805
MYOX00000000	SRR1816503	BCW_4895	*S. enterica*		80	4,766,604
MYOW00000000	SRR1816504	BCW_4896	*S. enterica*		61	4,702,091
MYOV00000000	SRR1816505	BCW_4897	*S. enterica*		64	4,510,094
MYOU00000000	SRR1816506	BCW_4898	*S. enterica*		39	4,854,041
MYOT00000000	SRR1816507	BCW_4899	*S. enterica*		75	4,929,027
MYOS00000000	SRR1816508	BCW_4900	*S. enterica*		52	4,788,114
MYOR00000000	SRR1816509	BCW_4901	*S. enterica*		48	4,638,809
MYOQ00000000	SRR1816510	BCW_4902	*S. enterica*		98	4,845,443
MYOP00000000	SRR1816511	BCW_4903	*S. enterica*		39	4,665,184
MYOO00000000	SRR1816512	BCW_4904	*S. enterica*		72	4,861,097
MYON00000000	SRR1816513	BCW_4905	*S. enterica*		57	4,826,391
MYOM00000000	SRR1816514	BCW_4906	*S. enterica*		61	4,709,512
MYOL00000000	SRR1816515	BCW_4907	*S. enterica*		81	4,748,268
MYOK00000000	SRR1816516	BCW_4908	*S. enterica*		53	4,617,710
MYOJ00000000	SRR1816518	BCW_4910	*S. enterica*		65	4,699,642
MYOI00000000	SRR1816519	BCW_4911	*S. enterica*		95	4,888,754
MYOH00000000	SRR1816520	BCW_4912	*S. enterica*		77	4,677,955
MYOG00000000	SRR1816521	BCW_4913	*S. enterica*		83	4,830,988
MYOF00000000	SRR1816522	BCW_4914	*S. enterica*		64	4,830,410
MYOE00000000	SRR1816523	BCW_4915	*S. enterica*		42	4,653,833
MYOD00000000	SRR1816524	BCW_4916	*S. enterica*		183	4,760,274
MYOC00000000	SRR1816525	BCW_4917	*S. enterica*		37	4,752,300
MYOB00000000	SRR1816526	BCW_4918	*S. enterica*		72	4,802,006
MYOA00000000	SRR1816527	BCW_4919	*S. enterica*		28	4,696,876
MYNZ00000000	SRR1816528	BCW_4920	*S. enterica*		78	4,904,746
MYNY00000000	SRR1816529	BCW_4921	*S. enterica*		61	4,690,844
MYNX00000000	SRR1816530	BCW_4922	*S. enterica*		67	4,927,613
MYNW00000000	SRR1816531	BCW_4923	*S. enterica*		71	4,904,606
MYNV00000000	SRR1816532	BCW_4924	*S. enterica*		69	4,904,147
MYNU00000000	SRR1816533	BCW_4925	*S. enterica*		78	4,688,736
MYNT00000000	SRR1816534	BCW_4926	*S. enterica*		64	4,669,011
MYNS00000000	SRR1816535	BCW_4927	*S. enterica*		51	4,787,809
MYNR00000000	SRR1816536	BCW_4928	*S. enterica*		98	4,843,888
MYNQ00000000	SRR1816537	BCW_4929	*S. enterica*		52	4,620,424
MYNP00000000	SRR1816538	BCW_4930	*S. enterica*		65	4,757,293
MYNO00000000	SRR1816539	BCW_4931	*S. enterica*		79	4,807,107
MYNN00000000	SRR1816541	BCW_4933	*S. enterica*		74	4,629,919
MYNM00000000	SRR1816542	BCW_4934	*S. enterica*		57	4,670,434
MYNL00000000	SRR1816543	BCW_4935	*S. enterica*		33	4,667,921
MYNK00000000	SRR1816544	BCW_4936	*S. enterica*		88	4,873,401
MYNJ00000000	SRR1816545	BCW_4937	*S. enterica*		35	4,708,860
MYNI00000000	SRR1816546	BCW_4938	*S. enterica*		31	4,705,215
MYNH00000000	SRR1816547	BCW_4939	*S. enterica*		65	4,715,137
MYNG00000000	SRR1816548	BCW_4940	*S. enterica*		67	4,606,750
MYNF00000000	SRR1816549	BCW_4941	*S. enterica*		71	4,752,673
MYNC00000000	SRR1816552	BCW_4944	*S. enterica*		76	4,656,228
MYMO00000000	SRR1816566	BCW_4958	*S. enterica*		54	4,953,576
MYMH00000000	SRR1816573	BCW_4986	*S. enterica*		243	4,769,308
MYME00000000	SRR1816576	BCW_4989	*S. enterica*		298	5,142,517
MYMB00000000	SRR1816582	BCW_5009	*S. enterica*		106	4,562,891
MYLZ00000000	SRR1816584	BCW_5011	*S. enterica*		102	4,946,220
MYLX00000000	SRR1816588	BCW_5037	*S. enterica*		81	4,772,512
MYLW00000000	SRR1816589	BCW_5038	*S. enterica*		53	4,670,024
MYLV00000000	SRR1816590	BCW_5039	*S. enterica*		193	4,835,257
MYLT00000000	SRR1816592	BCW_5041	*S. enterica*		177	4,886,915
MYLR00000000	SRR1816597	BCW_5047	*S. enterica*		254	4,871,718
MYLQ00000000	SRR1816598	BCW_5048	*S. enterica*		128	4,679,123
MYLO00000000	SRR1816600	BCW_5050	*S. enterica*		42	4,505,872
MYKN00000000	SRR1816632	BCW_5105	*S. enterica*		75	4,838,827
MYKM00000000	SRR1816633	BCW_5106	*S. enterica*		73	5,072,429
MYKL00000000	SRR1816634	BCW_5107	*S. enterica*		37	4,486,278
MYKK00000000	SRR1816635	BCW_5108	*S. enterica*		33	4,506,576
MYKJ00000000	SRR1816636	BCW_5109	*S. enterica*		77	4,791,805
MYKI00000000	SRR1816637	BCW_5110	*S. enterica*		41	4,918,738
MYKC00000000	SRR1816672	BCW_5227	*S. enterica*	Schwarzengrund	230	4,543,616
MYJU00000000	SRR1816680	BCW_5262	*S. enterica*		109	4,887,637
MYJN00000000	SRR1814858	BCW_5827	*S. enterica*	4,12:i:-	54	4,546,998
MYJM00000000	SRR1814859	BCW_5828	*S. enterica*	Typhimurium	77	4,806,285
MYJK00000000	SRR1814861	BCW_5830	*S. enterica*	1,4,12:-:1,2	82	4,763,992
MYJI00000000	SRR1814863	BCW_5832	*S. enterica*	Typhimurium	72	4,862,012
MYJH00000000	SRR1814864	BCW_5833	*S. enterica*	Typhimurium	161	4,939,658
MYJG00000000	SRR1814865	BCW_5834	*S. enterica*	Typhimurium	86	4,865,069
MYJF00000000	SRR1814866	BCW_5835	*S. enterica*	Typhimurium	153	4,859,254
MYJD00000000	SRR1814868	BCW_5837	*S. enterica*	4,12:i:-	161	5,153,574
MYJC00000000	SRR1814869	BCW_5838	*S. enterica*	4,[5],12:i:-	115	5,028,359
MYJB00000000	SRR1814870	BCW_5839	*S. enterica*	4,12:i:-	126	4,950,031
MYJA00000000	SRR1814871	BCW_5840	*S. enterica*	4,[5],12:i:-	130	4,920,671
MYIZ00000000	SRR1814872	BCW_5841	*S. enterica*	4,[5],12:i:-	132	5,201,459
MYIX00000000	SRR1814874	BCW_5843	*S. enterica*	4,12:i:-	96	4,917,937
MYIW00000000	SRR1814875	BCW_5844	*S. enterica*	4,[5],12:i:-	79	4,827,920
MYIV00000000	SRR1814876	BCW_5845	*S. enterica*	Typhimurium	88	4,896,433
MYIT00000000	SRR1814878	BCW_5847	*S. enterica*	4,[5],12:i:-	128	4,858,094
MYIS00000000	SRR1814879	BCW_5848	*S. enterica*	Typhimurium	124	4,907,805
MYIP00000000	SRR1814882	BCW_5851	*S. enterica*	4,12:i:-	99	4,900,685
MYIN00000000	SRR1814884	BCW_5853	*S. enterica*	Enteritidis	55	4,736,047
MYIM00000000	SRR1814885	BCW_5854	*S. enterica*	Enteritidis	60	4,707,849
MYIL00000000	SRR1814886	BCW_5855	*S. enterica*	4,[5],12:i:-	153	4,869,475
MYIJ00000000	SRR1814888	BCW_5857	*S. enterica*	4,[5],12:i:-	144	4,960,392
MYIH00000000	SRR1814890	BCW_5859	*S. enterica*	Typhimurium	111	5,155,971
MYIE00000000	SRR1814894	BCW_5863	*S. enterica*	4,12:i:-	147	5,036,041
MYIA00000000	SRR1814899	BCW_5868	*S. enterica*	Typhimurium	78	4,987,666
MYHZ00000000	SRR1814900	BCW_5870	*S. enterica*	Typhimurium	132	4,932,046
MYHY00000000	SRR1814901	BCW_5871	*S. enterica*	Typhimurium	102	4,881,675
MYHW00000000	SRR1814904	BCW_5874	*S. enterica*	Typhimurium	117	4,998,131
MYHV00000000	SRR1814905	BCW_5875	*S. enterica*	Typhimurium	211	4,843,570
MYHU00000000	SRR1814906	BCW_5876	*S. enterica*	Typhimurium	195	4,960,978
MYHT00000000	SRR1814907	BCW_5877	*S. enterica*	Typhimurium	115	4,972,933
MYHR00000000	SRR1814909	BCW_5879	*S. enterica*	Typhimurium	183	5,005,747
MYHQ00000000	SRR1814910	BCW_5880	*S. enterica*	Typhimurium	119	5,029,247
MYHP00000000	SRR1814911	BCW_5881	*S. enterica*	Typhimurium	109	4,973,403
MYHO00000000	SRR1814912	BCW_5882	*S. enterica*	Typhimurium	94	4,789,503
MYHN00000000	SRR1814913	BCW_5883	*S. enterica*	Typhimurium	85	4,948,763
MYHM00000000	SRR1814914	BCW_5884	*S. enterica*	Typhimurium	119	4,950,319
MYHI00000000	SRR1814918	BCW_5888	*S. enterica*	Typhimurium	111	4,969,814
MYHH00000000	SRR1814919	BCW_5889	*S. enterica*	Typhimurium	116	4,907,806
MYHG00000000	SRR1814920	BCW_5890	*S. enterica*	Typhimurium	112	4,939,849
MYHF00000000	SRR1814921	BCW_5891	*S. enterica*	Typhimurium	123	4,932,739
MYHE00000000	SRR1814922	BCW_5892	*S. enterica*	Typhimurium	90	4,870,931
MYHD00000000	SRR1814923	BCW_5893	*S. enterica*	Typhimurium	211	4,971,346
MYHB00000000	SRR1814925	BCW_5895	*S. enterica*	Typhimurium	135	4,933,960
MYGZ00000000	SRR1814927	BCW_5897	*S. enterica*	Enteritidis	202	4,757,626
MYGY00000000	SRR1814928	BCW_5898	*S. enterica*	Typhimurium	129	4,916,058
MYGX00000000	SRR1814929	BCW_5899	*S. enterica*	Typhimurium	111	4,991,400
MYGW00000000	SRR1814930	BCW_5900	*S. enterica*	Typhimurium	109	4,946,012
MYGV00000000	SRR1814931	BCW_5901	*S. enterica*	Typhimurium	70	4,985,024
MYGU00000000	SRR1814932	BCW_5902	*S. enterica*	Typhimurium	92	4,890,667
MYGT00000000	SRR1814933	BCW_5903	*S. enterica*	Indiana	81	4,695,154
MYGS00000000	SRR1814934	BCW_5904	*S. enterica*	Indiana	79	4,693,070
MYGR00000000	SRR1814935	BCW_5905	*S. enterica*	Montevideo	89	4,651,553
MYGQ00000000	SRR1814936	BCW_5906	*S. enterica*	Enteritidis	50	4,703,102
MYGP00000000	SRR1814937	BCW_5907	*S. enterica*	Indiana	214	5,000,185
MYGO00000000	SRR1814938	BCW_5908	*S. enterica*	Montevideo	135	4,735,317
MYGN00000000	SRR1814940	BCW_5910	*S. enterica*	Montevideo	45	4,705,549
MYGM00000000	SRR1814941	BCW_5911	*S. enterica*	Derby	99	4,706,266
MYGL00000000	SRR1753633	BCW_5935	*S. enterica*	Bovismorbificans	70	4,732,903
MYGJ00000000	SRR1753634	BCW_5937	*S. enterica*	Weltevreden	174	5,040,992
MYGI00000000	SRR1753635	BCW_5938	*S. enterica*	Muenster	55	4,629,211
MYGG00000000	SRR1753637	BCW_5940	*S. enterica*	Kentucky	158	5,150,542
MYGF00000000	SRR1753638	BCW_5941	*S. enterica*	Miami	90	4,834,722
MYGE00000000	SRR1753639	BCW_5942	*S. enterica*	Muenster	81	4,854,793
MYGD00000000	SRR1753640	BCW_5947	*S. enterica*	Miami	195	4,580,816
MYGB00000000	SRR1753641	BCW_5949	*S. enterica*	Kentucky	82	4,717,419
MYGA00000000	SRR1753642	BCW_5950	*S. enterica*	Weltevreden	161	5,208,346
MYFZ00000000	SRR1753643	BCW_5951	*S. enterica*	Gaminara	52	4,538,913
MYFY00000000	SRR1753644	BCW_5952	*S. enterica*	Bovismorbificans	58	4,605,133
MYFX00000000	SRR1753645	BCW_5953	*S. enterica*	Muenster	47	4,718,036
MYFW00000000	SRR1753646	BCW_5954	*S. enterica*	Miami	75	4,616,722
MYFU00000000	SRR1753648	BCW_5956	*S. enterica*	Muenster	37	4,593,200
MYFT00000000	SRR1753649	BCW_5957	*S. enterica*	Miami	54	4,580,606
MYFS00000000	SRR1753650	BCW_5958	*S. enterica*	Kentucky	71	4,722,160
MYFR00000000	SRR1753651	BCW_5959	*S. enterica*	Weltevreden	111	4,968,419
MZGF00000000	SRR1753652	BCW_5960	*S. enterica*	Gaminara	48	4,541,461
MZGD00000000	SRR1753654	BCW_5962	*S. enterica*	Gaminara	92	5,093,951
MZGC00000000	SRR1753655	BCW_5964	*S. enterica*	Miami	71	4,581,425
MZGB00000000	SRR1753656	BCW_5966	*S. enterica*	Virchow	54	4,491,231
MZGA00000000	SRR1753657	BCW_5967	*S. enterica*	Kentucky	58	4,776,051
MZFZ00000000	SRR1753658	BCW_5968	*S. enterica*	Gaminara	49	4,561,851
MZFY00000000	SRR1753660	BCW_5971	*S. enterica*	Bovismorbificans	37	4,688,243
MZFX00000000	SRR1753661	BCW_5973	*S. enterica*	Weltevreden	111	5,016,671
MZFW00000000	SRR1753662	BCW_5974	*S. enterica*	Virchow	52	4,680,614
MZFV00000000	SRR1753663	BCW_5975	*S. enterica*	Blockley	249	5,575,838
MZFU00000000	SRR1753664	BCW_5976	*S. enterica*	Adelaide	29	4,560,267
MZFT00000000	SRR1753665	BCW_5977	*S. enterica*	Ohio	26	4,850,583
MZFS00000000	SRR1753666	BCW_5978	*S. enterica*	Pomona	62	4,451,058
MZFR00000000	SRR1753667	BCW_5979	*S. enterica*	Adelaide	51	4,493,063
MZFQ00000000	SRR1814990	BCW_5980	*S. enterica*	Dublin	32	4,675,704
MZFP00000000	SRR1753668	BCW_5981	*S. enterica*	Ohio	73	4,827,114
MZFO00000000	SRR1753669	BCW_5982	*S. enterica*	Pomona	42	4,451,338
MZFM00000000	SRR1753671	BCW_5984	*S. enterica*	Reading	79	4,832,466
MZFL00000000	SRR1753672	BCW_5985	*S. enterica*	Adelaide	37	4,690,913
MZFK00000000	SRR1753673	BCW_5986	*S. enterica*	Reading	90	4,542,190
MZFJ00000000	SRR1753674	BCW_5987	*S. enterica*	Ohio	69	4,689,964
MZFI00000000	SRR1814998	BCW_5988	*S. enterica*	Pomona	60	4,755,333
MZFH00000000	SRR1814999	BCW_5989	*S. enterica*	Blockley	40	4,448,104
MZFF00000000	SRR1753675	BCW_5991	*S. enterica*	Pomona	37	4,412,782
MZFE00000000	SRR1753676	BCW_5992	*S. enterica*	Ohio	63	4,696,650
MZFD00000000	SRR1753677	BCW_5993	*S. enterica*	Adelaide	50	4,495,470
MZFC00000000	SRR1753678	BCW_5994	*S. enterica*	Blockley	45	4,869,614
MZFB00000000	SRR1753679	BCW_5997	*S. enterica*	Pomona	50	4,728,423
MZFA00000000	SRR1753680	BCW_5998	*S. enterica*	Manhattan	60	4,643,018
MZEZ00000000	SRR1753681	BCW_5999	*S. enterica*	Blockley	45	4,729,445
MZEX00000000	SRR1753682	BCW_6001	*S. enterica*	Minnesota	38	4,585,895
MZEW00000000	SRR1753683	BCW_6002	*S. enterica*	Inverness	43	4,998,352
MZEV00000000	SRR1753684	BCW_6003	*S. enterica*	Kiambu	48	4,483,229
MZEU00000000	SRR1753685	BCW_6004	*S. enterica*	Uganda	49	4,694,602
MZET00000000	SRR1753686	BCW_6005	*S. enterica*	Urbana	158	4,993,666
MZES00000000	SRR1753687	BCW_6006	*S. enterica*	Hvittingfoss	38	4,666,586
MZER00000000	SRR1815015	BCW_6007	*S. enterica*	Hvittingfoss	43	4,867,072
MZEQ00000000	SRR1753688	BCW_6008	*S. enterica*	Minnesota	57	4,595,541
MZEP00000000	SRR1753689	BCW_6009	*S. enterica*	I 4,5,12:b:-	157	4,797,117
MZEO00000000	SRR1753690	BCW_6010	*S. enterica*	Inverness	52	4,807,994
MZEN00000000	SRR1753691	BCW_6011	*S. enterica*	Kiambu	34	4,480,561
MZEM00000000	SRR1753692	BCW_6012	*S. enterica*	Uganda	80	4,803,514
MZEK00000000	SRR1753694	BCW_6014	*S. enterica*	Minnesota	65	4,712,253
MZEI00000000	SRR1753695	BCW_6016	*S. enterica*	Urbana	118	5,018,992
MZEH00000000	SRR1753696	BCW_6017	*S. enterica*	I 4,5,12:b:-	75	4,736,751
MZEG00000000	SRR1753697	BCW_6018	*S. enterica*	Hvittingfoss	44	4,714,829
MZEF00000000	SRR1753698	BCW_6019	*S. enterica*	Inverness	68	5,050,709
MZEE00000000	SRR1753699	BCW_6020	*S. enterica*	Urbana	120	4,957,372
MZED00000000	SRR1815029	BCW_6021	*S. enterica*	Kiambu	58	4,639,045
MZEC00000000	SRR1753700	BCW_6022	*S. enterica*	Hvittingfoss	108	4,673,668
MZEB00000000	SRR1753701	BCW_6023	*S. enterica*	Uganda	62	4,784,075
MZEA00000000	SRR1753702	BCW_6024	*S. enterica*	I 4,12:b:-	32	4,808,859
MZDY00000000	SRR1753704	BCW_6026	*S. enterica*	Kiambu	150	4,509,398
MZDX00000000	SRR1753705	BCW_6027	*S. enterica*	Inverness	157	4,964,713
MZDW00000000	SRR1753707	BCW_6030	*S. enterica*	Uganda	147	4,668,843
MZDV00000000	SRR1753708	BCW_6031	*S. enterica*	Minnesota	179	4,633,156
MZDT00000000	SRR1753710	BCW_6034	*S. enterica*	Hvittingfoss	58	4,695,779
MZDS00000000	SRR1753711	BCW_6035	*S. enterica*	Urbana	39	4,726,844
MZDR00000000	SRR1753712	BCW_6036	*S. enterica*	Minnesota	83	4,591,733
MZDQ00000000	SRR1753713	BCW_6037	*S. enterica*	Havana	70	4,859,290
MZDO00000000	SRR1753715	BCW_6039	*S. enterica*	Telelkebir	68	4,660,002
MZDN00000000	SRR1753716	BCW_6040	*S. enterica*	IV 44:z4,z23:-	97	4,597,275
MZDM00000000	SRR1753717	BCW_6041	*S. enterica*	Bredeney	57	4,737,947
MZDL00000000	SRR1753718	BCW_6042	*S. enterica*	Cerro	74	4,676,865
MZDK00000000	SRR1753719	BCW_6043	*S. enterica*	Telelkebir	75	4,767,998
MZDJ00000000	SRR1753720	BCW_6044	*S. enterica*	Bredeney	53	4,649,459
MZDI00000000	SRR1753721	BCW_6045	*S. enterica*	London	77	4,973,779
MZDH00000000	SRR1753722	BCW_6046	*S. enterica*	Telelkebir	63	4,568,808
MZDG00000000	SRR1753723	BCW_6047	*S. enterica*	Havana	65	4,805,815
MZDF00000000	SRR1753724	BCW_6049	*S. enterica*	Cerro	83	4641746
MZDE00000000	SRR1753725	BCW_6050	*S. enterica*	Bredeney	45	4,588,214
MZDD00000000	SRR1753726	BCW_6051	*S. enterica*	Johannesburg	82	4,677,641
MZDC00000000	SRR1753727	BCW_6052	*S. enterica*	Telelkebir	64	4,607,374
MZDB00000000	SRR1753728	BCW_6053	*S. enterica*	Johannesburg	98	4,826,945
MZDA00000000	SRR1753729	BCW_6054	*S. enterica*	Bredeney	53	4,821,832
MZCZ00000000	SRR1753730	BCW_6055	*S. enterica*	Havana	70	4,943,752
MZCY00000000	SRR1753731	BCW_6056	*S. enterica*	Newport	221	5,063,691
MZCX00000000	SRR1753732	BCW_6057	*S. enterica*	Bredeney	53	4,674,653
MZCW00000000	SRR1753733	BCW_6058	*S. enterica*	Bredeney	35	4,634,860
MZCV00000000	SRR1753734	BCW_6059	*S. enterica*	Telelkebir	68	4,613,740
MZCU00000000	SRR1753735	BCW_6060	*S. enterica*	Telelkebir	132	5,032,061
MZCT00000000	SRR1815066	BCW_6061	*S. enterica*	Cerro	53	4,615,726
MZCS00000000	SRR1815067	BCW_6062	*S. enterica*	Johannesburg	75	4,799,209
MZCR00000000	SRR1753736	BCW_6063	*S. enterica*	Johannesburg	42	4,567,118
MZCQ00000000	SRR1753737	BCW_6064	*S. enterica*	Cerro	71	4,548,490
MZCP00000000	SRR1753738	BCW_6065	*S. enterica*	London	95	4,749,277
MZCO00000000	SRR1753740	BCW_6067	*S. enterica*	Havana	214	4,652,566
MZCL00000000	SRR1753743	BCW_6070	*S. enterica*	Grumpensis	60	4,533,267
MZCK00000000	SRR1753744	BCW_6071	*S. enterica*	Albany	123	4,770,257
MZCJ00000000	SRR1753745	BCW_6072	*S. enterica*	Worthington	75	5,088,834
MZCI00000000	SRR1753746	BCW_6073	*S. enterica*	Chester	54	4,752,112
MZCH00000000	SRR1753747	BCW_6074	*S. enterica*	Albany	51	4,810,861
MZCG00000000	SRR1753748	BCW_6075	*S. enterica*	Oslo	230	4,551,393
MZCF00000000	SRR1753749	BCW_6076	*S. enterica*	Indiana	125	4,821,947
MZCE00000000	SRR1753750	BCW_6077	*S. enterica*	Grumpensis	195	4,595,797
MZCD00000000	SRR1753751	BCW_6078	*S. enterica*	Chester	64	4,740,601
MZBZ00000000	SRR1815087	BCW_6082	*S. enterica*	Agbeni	51	4,785,183
MZBW00000000	SRR1753757	BCW_6085	*S. enterica*	Worthington	53	4,775,647
MZBV00000000	SRR1753759	BCW_6087	*S. enterica*	Chester	46	4,652,040
MZBU00000000	SRR1753760	BCW_6088	*S. enterica*	Oslo	60	4,924,504
MZBT00000000	SRR1815094	BCW_6089	*S. enterica*	Agbeni	66	4,833,217
MZBS00000000	SRR1753761	BCW_6090	*S. enterica*	Oslo	43	4,546,183
MZBR00000000	SRR1753762	BCW_6091	*S. enterica*	Indiana	234	4,760,739
MZBQ00000000	SRR1753763	BCW_6092	*S. enterica*	Agbeni	66	4,676,150
MZBP00000000	SRR1753764	BCW_6093	*S. enterica*	Chester	124	4,641,115
MZBO00000000	SRR1753765	BCW_6094	*S. enterica*	Worthington	70	4,865,820
MZBN00000000	SRR1753766	BCW_6095	*S. enterica*	Chester	60	4,866,135
MZBM00000000	SRR1753767	BCW_6096	*S. enterica*	Indiana	77	4,898,026
MZBL00000000	SRR1753768	BCW_6097	*S. enterica*	Indiana	74	4,739,393
MZBJ00000000	SRR1753772	BCW_6101	*S. enterica*	Grumpensis	102	4,549,752
MZBI00000000	SRR1753773	BCW_6102	*S. enterica*	Grumpensis	88	4,601,152
MZBH00000000	SRR1753774	BCW_6103	*S. enterica*	Worthington	66	4,914,760
MZBG00000000	SRR1753775	BCW_6104	*S. enterica*	Albany	41	4,727,611
MZBF00000000	SRR1753776	BCW_6105	*S. enterica*	Albany	62	4,799,691
MZBE00000000	SRR1753777	BCW_6106	*S. enterica*	Agbeni	66	4,591,561
MZBD00000000	SRR1753778	BCW_6107	*S. enterica*	Loma Linda	87	4,584,522
MZBC00000000	SRR1753779	BCW_6108	*S. enterica*	Ealing	75	4,798,099
MZBB00000000	SRR1753780	BCW_6109	*S. enterica*	IV 48:g,z51:-	72	4,759,948
MZBA00000000	SRR1753781	BCW_6110	*S. enterica*	Cubana	105	4,971,518
MZAZ00000000	SRR1753782	BCW_6111	*S. enterica*	Edinburg	74	4,545,932
MZAY00000000	SRR1753783	BCW_6112	*S. enterica*	Eastbourne	47	4,662,422
MZAX00000000	SRR1753784	BCW_6113	*S. enterica*	Alachua	32	4,817,049
MZAW00000000	SRR1753785	BCW_6114	*S. enterica*	Baildon	61	4,687,449
MZAU00000000	SRR1753788	BCW_6117	*S. enterica*	Loma Linda	102	4,581,157
MZAT00000000	SRR1753789	BCW_6118	*S. enterica*	Alachua	56	4,844,382
MZAS00000000	SRR1753790	BCW_6119	*S. enterica*	Baildon	59	4,634,502
MZAR00000000	SRR1815125	BCW_6120	*S. enterica*	Eastbourne	252	5,197,359
MZAQ00000000	SRR1753791	BCW_6121	*S. enterica*	IV 50:z4,z23:-	115	5,024,314
MZAP00000000	SRR1753792	BCW_6122	*S. enterica*	Edinburg	80	4,722,319
MZAO00000000	SRR1753793	BCW_6123	*S. enterica*	IV 48:g,z51:-	88	4,660,603
MZAN00000000	SRR1753794	BCW_6124	*S. enterica*	IV 50:z4,z23:-	93	4,930,341
MZAL00000000	SRR1753796	BCW_6126	*S. enterica*	Baildon	70	4,838,330
MZAK00000000	SRR1753797	BCW_6127	*S. enterica*	IV 48:g,z51:-	105	4,622,285
MZAJ00000000	SRR1815133	BCW_6128	*S. enterica*	Alachua	48	4,515,165
MZAI00000000	SRR1753798	BCW_6129	*S. enterica*	Cubana	57	4,889,759
MZAH00000000	SRR1753799	BCW_6130	*S. enterica*	Loma Linda	91	4,618,678
MZAF00000000	SRR1753801	BCW_6133	*S. enterica*	Loma Linda	188	4,574,002
MZAE00000000	SRR1753802	BCW_6134	*S. enterica*	Cubana	93	4,900,616
MZAD00000000	SRR1753803	BCW_6135	*S. enterica*	Eastbourne	55	4,666,773
MZAC00000000	SRR1753804	BCW_6136	*S. enterica*	Baildon	93	4,862,028
MZAA00000000	SRR1753805	BCW_6139	*S. enterica*	Alachua	64	4,862,440
MYZZ00000000	SRR1753806	BCW_6140	*S. enterica*	Edinburg	78	4,522,056
MYZY00000000	SRR1753807	BCW_6141	*S. enterica*	IV 48:g,z51:-	92	4,581,026
MYZW00000000	SRR1753809	BCW_6143	*S. enterica*	Loma Linda	89	4,576,396
MYZV00000000	SRR1753810	BCW_6144	*S. enterica*	Eastbourne	90	4,371,556
MYZU00000000	SRR1753811	BCW_6145	*S. enterica*	Alachua	75	4,924,790
MYZT00000000	SRR1753812	BCW_6146	*S. enterica*	Loma Linda	85	4,579,090
MYZS00000000	SRR1753813	BCW_6147	*S. enterica*	Eastbourne	52	4,770,658
MYZR00000000	SRR1753814	BCW_6148	*S. enterica*	Blockley	59	4,833,575
MYZQ00000000	SRR1753815	BCW_6149	*S. enterica*	IV 50:z4,z23:-	130	4,598,225
MYZP00000000	SRR1753816	BCW_6151	*S. enterica*	Cubana	86	4,900,289
MYZO00000000	SRR1753817	BCW_6152	*S. enterica*	IV 50:z4,z23:-	88	4,572,619
MYZN00000000	SRR1753818	BCW_6153	*S. enterica*	Ealing	42	4,725,736
MYZL00000000	SRR1753820	BCW_6155	*S. enterica*	Cubana	68	4,874,989
MYZK00000000	SRR1753821	BCW_6156	*S. enterica*	Blockley	46	4,703,470
MYZJ00000000	SRR1753822	BCW_6157	*S. enterica*	Baildon	54	4,632,376
MYZI00000000	SRR1753823	BCW_6158	*S. enterica*	Baildon	75	4,671,812
MYZG00000000	SRR1753824	BCW_6160	*S. enterica*	Meleagridis	49	4,765,153
MYZF00000000	SRR1753825	BCW_6161	*S. enterica*	I 9,12:l,z28:-	29	4,664,275
MYZE00000000	SRR1753826	BCW_6162	*S. enterica*	IV 50:g,z51:-	146	4,789,405
MYZD00000000	SRR1753827	BCW_6163	*S. enterica*	Choleraesuis var. Kunzendorf	72	4,737,676
MYZC00000000	SRR1753828	BCW_6164	*S. enterica*	Kintambo	97	4,860,358
MYZB00000000	SRR1753829	BCW_6165	*S. enterica*	IV 44:z4,z23:-	67	4,590,893
MYZA00000000	SRR1753830	BCW_6166	*S. enterica*	Kottbus	54	4,662,259
MYYZ00000000	SRR1815170	BCW_6167	*S. enterica*	I 9,12:l,z28:-	70	4,488,294
MYYY00000000	SRR1753831	BCW_6168	*S. enterica*	Ibadan	34	4,522,045
MYYX00000000	SRR1753832	BCW_6169	*S. enterica*	Meleagridis	45	4,795,066
MYYW00000000	SRR1753833	BCW_6170	*S. enterica*	Monschaui	49	4,892,392
MYYV00000000	SRR1753834	BCW_6171	*S. enterica*	IV 44:z4,z23:-	44	4,605,738
MYYU00000000	SRR1753835	BCW_6172	*S. enterica*	Kintambo	106	4,972,684
MYYT00000000	SRR1753836	BCW_6173	*S. enterica*	Choleraesuis var. Kunzendorf	92	4,737,938
MYYS00000000	SRR1753837	BCW_6174	*S. enterica*	Corvallis	60	4,804,015
MYYR00000000	SRR1753838	BCW_6175	*S. enterica*	IV 50:g,z51:-	130	4,841,611
MYYQ00000000	SRR1753839	BCW_6176	*S. enterica*	Rissen	66	4,832,259
MYYP00000000	SRR1753840	BCW_6177	*S. enterica*	Kottbus	56	4,736,546
MYYO00000000	SRR1753841	BCW_6178	*S. enterica*	Corvallis	45	4,625,804
MYYN00000000	SRR1753842	BCW_6179	*S. enterica*	Monschaui	53	4,809,938
MYYM00000000	SRR1753843	BCW_6180	*S. enterica*	IV 50:g,z51:-	93	4,676,163
MYYL00000000	SRR1753844	BCW_6181	*S. enterica*	IV 44:z4,z23:-	85	4,559,520
MYYK00000000	SRR1753845	BCW_6182	*S. enterica*	Meleagridis	44	4,795,704
MYYJ00000000	SRR1815186	BCW_6183	*S. enterica*	Kintambo	198	4,834,694
MYYI00000000	SRR1753848	BCW_6186	*S. enterica*	I 9,12:l,z28:-	53	4,563,716
MYYH00000000	SRR1753849	BCW_6187	*S. enterica*	Rissen	87	4,945,922
MYYG00000000	SRR1753850	BCW_6188	*S. enterica*	Kintambo	81	4,807,235
MYYF00000000	SRR1753851	BCW_6189	*S. enterica*	Kottbus	38	4,756,012
MYYE00000000	SRR1753852	BCW_6191	*S. enterica*	Meleagridis	61	4,829,317
MYYD00000000	SRR1753853	BCW_6192	*S. enterica*	IV 44:z4,z23:-	90	4,613,029
MYYC00000000	SRR1753854	BCW_6193	*S. enterica*	Choleraesuis	87	4,741,704
MYYB00000000	SRR1753855	BCW_6194	*S. enterica*	Choleraesuis var. Kunzendorf	91	4,822,344
MYYA00000000	SRR1753856	BCW_6195	*S. enterica*	Monschaui	197	5,061,534
MYXZ00000000	SRR1753857	BCW_6196	*S. enterica*	Meleagridis	42	4,719,812
MYXX00000000	SRR1753859	BCW_6200	*S. enterica*	IV 50:g,z51:-	115	4,761,962
MYXW00000000	SRR1753860	BCW_6201	*S. enterica*	Monschaui	64	4,740,104
MYXU00000000	SRR1815213	BCW_6222	*S. enterica*	Derby	96	4,897,224
MYXT00000000	SRR1815214	BCW_6223	*S. enterica*	Derby	128	4,896,999
MYXS00000000	SRR1815215	BCW_6224	*S. enterica*	München	214	5,272,810
MYXR00000000	SRR1815216	BCW_6225	*S. enterica*	München	84	5,265,120
MYXQ00000000	SRR1815217	BCW_6226	*S. enterica*	München	92	5,256,870
MYXP00000000	SRR1815218	BCW_6227	*S. enterica*	Derby	150	4,921,818
MYXN00000000	SRR1815220	BCW_6229	*S. enterica*	München	84	5,136,972
MYXM00000000	SRR1815223	BCW_6232	*S. enterica*	München	71	5,137,398
MYXL00000000	SRR1815224	BCW_6233	*S. enterica*	München	137	5,147,066
MYXK00000000	SRR1815225	BCW_6234	*S. enterica*	München	75	5,137,903
MYXJ00000000	SRR1815226	BCW_6235	*S. enterica*	München	42	4,528,277
MYXI00000000	SRR1815227	BCW_6236	*S. enterica*	Senftenberg	95	4,779,616
MYXH00000000	SRR1815228	BCW_6237	*S. enterica*	Senftenberg	111	4,784,679
MYXG00000000	SRR1815229	BCW_6238	*S. enterica*	Senftenberg	70	4,815,347
MYXD00000000	SRR1815232	BCW_6241	*S. enterica*	Derby	58	4,813,148
MYXC00000000	SRR1815233	BCW_6242	*S. enterica*	Derby	86	4,802,471
MYXB00000000	SRR1815235	BCW_6244	*S. enterica*	Mbandaka	79	4,884,522
MYXA00000000	SRR1815236	BCW_6245	*S. enterica*	Mbandaka	81	4,886,387
MYWZ00000000	SRR1815237	BCW_6247	*S. enterica*	Johannesburg	50	4,572,794
MYWX00000000	SRR1815240	BCW_6250	*S. enterica*	Johannesburg	49	4,571,673
MYWW00000000	SRR1815241	BCW_6251	*S. enterica*	III_44:z4,z32	107	4,496,041
MYWV00000000	SRR1815242	BCW_6252	*S. enterica*	III_44:z4,z33	155	4,495,655
MYWT00000000	SRR1815244	BCW_6254	*S. enterica*	München	60	5,122,463
MYWS00000000	SRR1815245	BCW_6255	*S. enterica*	München	52	5,123,499
MYWR00000000	SRR1815246	BCW_6256	*S. enterica*	München	106	5,150,227
MYWQ00000000	SRR1815247	BCW_6257	*S. enterica*	Braenderup	51	4,661,166
MYWO00000000	SRR1815249	BCW_6259	*S. enterica*	Braenderup	66	4,655,551
MYWN00000000	SRR1815250	BCW_6260	*S. enterica*	Johannesburg	69	4,800,375
MYWL00000000	SRR1815252	BCW_6262	*S. enterica*	Mbandaka	253	4,848,695
MYWK00000000	SRR1815253	BCW_6263	*S. enterica*	Mbandaka	77	4,853,598
MYWJ00000000	SRR1815255	BCW_6265	*S. enterica*	München	77	5,213,813
MYWI00000000	SRR1815256	BCW_6266	*S. enterica*	München	75	5,223,730
MYWH00000000	SRR1815257	BCW_6267	*S. enterica*	München	114	4,974,278
MYWG00000000	SRR1815258	BCW_6268	*S. enterica*	München	300	5,201,901
MYWF00000000	SRR1815259	BCW_6269	*S. enterica*	München	76	5,235,520
MYWE00000000	SRR1815260	BCW_6270	*S. enterica*	München	95	4,790,142
MYWD00000000	SRR1815261	BCW_6271	*S. enterica*	München	153	5,167,111
MYWC00000000	SRR1815262	BCW_6272	*S. enterica*	München	74	4,962,090
MYWB00000000	SRR1815263	BCW_6273	*S. enterica*	Derby	127	4,994,272
MYWA00000000	SRR1815264	BCW_6274	*S. enterica*	München	204	5,091,697
MYVX00000000	SRR1815267	BCW_6277	*S. enterica*	München	108	5,304,835
MYVU00000000	SRR1815270	BCW_6280	*S. enterica*	München	78	5,185,461
MYVS00000000	SRR1815272	BCW_6282	*S. enterica*	München	112	4,986,299
MYVR00000000	SRR1815273	BCW_6283	*S. enterica*	Derby	89	4,913,364
MYVP00000000	SRR1815275	BCW_6285	*S. enterica*	München	52	4,773,141
MYVM00000000	SRR1815278	BCW_6288	*S. enterica*	München	66	5,050,632
MYVK00000000	SRR1815280	BCW_6290	*S. enterica*	München	46	4,687,046
MYVJ00000000	SRR1815281	BCW_6291	*S. enterica*	Derby	73	4,808,341
MYVI00000000	SRR1815282	BCW_6292	*S. enterica*	Derby	66	4,799,051
MYVH00000000	SRR1815283	BCW_6293	*S. enterica*	Derby	85	4,854,721
MYVG00000000	SRR1815285	BCW_6295	*S. enterica*	München	94	4,733,351
MYVF00000000	SRR1815286	BCW_6296	*S. enterica*	München	66	5,299,186
MYVE00000000	SRR1815287	BCW_6297	*S. enterica*	München	38	4,613,440
MYVA00000000	SRR1815291	BCW_6301	*S. enterica*	Derby	87	4,914,839
MYUZ00000000	SRR1815292	BCW_6302	*S. enterica*	Mbandaka	58	4,822,164
MYUU00000000	SRR1815299	BCW_6309	*S. enterica*	Mbandaka	55	4,931,572
MYUT00000000	SRR1815370	BCW_6499	*S. enterica*	Montevideo	76	4,700,200
MYUS00000000	SRR1815371	BCW_6505	*S. enterica*	Heidelberg	71	4,539,985
MYUR00000000	SRR1815372	BCW_6510	*S. enterica*	Worthington	84	4,715,427
MYUQ00000000	SRR1815373	BCW_6524	*S. enterica*	Agona	42	4,589,330
MYUP00000000	SRR1815374	BCW_6525	*S. enterica*	Agona	64	4,649,648
MYUO00000000	SRR1815375	BCW_6526	*S. enterica*	Agona	66	4,651,722
MYUN00000000	SRR1815376	BCW_6532	*S. enterica*	Amsterdam	63	4,706,044
MYUM00000000	SRR1815378	BCW_6559	*S. enterica*	Dublin	61	4,882,116
MYUL00000000	SRR1815379	BCW_6571	*S. enterica*	Dublin	64	4,882,017
MYUK00000000	SRR1815380	BCW_6579	*S. enterica*	Dublin	93	4,972,587
MYUJ00000000	SRR1815381	BCW_6586	*S. enterica*	Anatum	33	4,669,928
MYUI00000000	SRR1815382	BCW_6599	*S. enterica*		42	4,933,066
MYUG00000000	SRR1815385	BCW_6620	*S. enterica*	Dublin	67	4,942,717
MYUF00000000	SRR1815386	BCW_6621	*S. enterica*	Dublin	62	4,944,002
MYUE00000000	SRR1815387	BCW_6623	*S. enterica*	Dublin	69	4,941,796
MYUD00000000	SRR1815391	BCW_6654	*S. enterica*	Dublin	98	4,896,266
MYUC00000000	SRR1815392	BCW_6659	*S. enterica*	Derby	38	4,877,491
